# Suprapapillary Biliary Stents Have Longer Patency Times than Transpapillary Stents—A Systematic Review and Meta-Analysis

**DOI:** 10.3390/jcm12030898

**Published:** 2023-01-23

**Authors:** Norbert Kovács, Dániel Pécsi, Zoltán Sipos, Nelli Farkas, Mária Földi, Péter Hegyi, Judit Bajor, Bálint Erőss, Katalin Márta, Alexandra Mikó, Zoltán Rakonczay, Patrícia Sarlós, Szabolcs Ábrahám, Áron Vincze

**Affiliations:** 1 Doctoral School of Clinical Medicine, University of Szeged, 6720 Szeged, Hungary; 2 Institute for Translational Medicine, Medical School, University of Pécs, 7624 Pécs, Hungary; 3 Centre for Translational Medicine, Semmelweis University, 1085 Budapest, Hungary; 4 Division of Gastroenterology, First Department of Medicine, Medical School, University of Pécs, 7624 Pécs, Hungary; 5 Institute of Bioanalysis, Medical School, University of Pécs, 7624 Pécs, Hungary; 6 Heim Pál National Pediatric Institute, 1089 Budapest, Hungary; 7 Institute of Pancreatic Diseases, Semmelweis University, 1083 Budapest, Hungary; 8 Translational Pancreatology Research Group, Interdisciplinary Centre of Excellence for Research Development and Innovation University of Szeged, 6725 Szeged, Hungary; 9 Department of Medical Genetics, Medical School, University of Pécs, 7624 Pécs, Hungary; 10 Department of Pathophysiology, University of Szeged, 6720 Szeged, Hungary; 11 Department of Surgery, Szent-Györgyi Albert Medical and Pharmaceutical Centre, University of Szeged, 6720 Szeged, Hungary

**Keywords:** stent, inside, intraductal, ERCP, endoscopy

## Abstract

Background and study aims: Endoscopic biliary stent placement is a minimally invasive intervention for patients with biliary strictures. Stent patency and function time are crucial factors. Suprapapillary versus transpapillary stent positioning may contribute to stent function time, so a meta-analysis was performed in this comparison. Methods: A comprehensive literature search was conducted in the CENTRAL, Embase, and MEDLINE databases to find data on suprapapillary stent placement compared to the transpapillary method via endoscopic retrograde cholangiopancreatography in cases of biliary stenosis of any etiology and any stent type until December 2020. We carried out a meta-analysis focusing on the following outcomes: stent patency, stent migration, rate of cholangitis and pancreatitis, and other reported complications. Results: Three prospective and ten retrospective studies involving 1028 patients were included. Suprapapillary stent placement appeared to be superior to transpapillary stent positioning in patency (weighted mean difference = 50.23 days, 95% CI: 8.56, 91.98; *p* = 0.0.018). In a subgroup analysis of malignant indications, suprapapillary positioning showed a lower rate of cholangitis (OR: 0.34, 95% CI: 0.13, 0.93; *p* = 0.036). Another subgroup analysis investigating metal stents in a suprapapillary position resulted in a lower rate of pancreatitis (OR: 0.16, 95% CI: 0.03, 0.95; *p* = 0.043) compared to transpapillary stent placement. There was no difference in stent migration rates between the two groups (OR: 0.67, 95% CI: 0.17, 2.72; *p* = 0.577). Conclusions: Based on our results, suprapapillary biliary stenting has longer stent patency. Moreover, the stent migration rate did not differ between the suprapapillary and transpapillary groups.

## 1. Introduction

Endoscopic biliary stent placement is a minimally invasive intervention for patients with biliary strictures of benign etiology [1] and for the palliative treatment of biliary malignancies to relieve symptoms and improve quality of life [2]. The most important issues in the endoscopic treatment of biliary obstruction are stent occlusion and stent patency time. The occlusion mechanism of biliary stents is still a subject of extensive research. Many factors, including stent position [3], diameter [4], material, and side holes, may play essential roles in stent occlusion [5,6]. Other factors, such as bacterial adherence to the stent wall and the deposition of dietary fibers into the stent lumen [7], likely contribute to stent clogging [8].

The standard method of biliary stent insertion is the so-called transpapillary stent (TPS) position or the ‘through the Oddi sphincter technique’. In this case, the stent crosses the papilla and the sphincter of Oddi (SO). The end of the stent protrudes into the duodenal lumen, thus facilitating its removal; it is also theoretically less likely to proximally migrate [8]. Another way to insert a biliary stent is to place the distal end of the stent above the SO within the common bile duct; this is consequently called the suprapapillary stent (SPS) position. In theory, when using this approach, the protection of the sphincter could lead to fewer stent occlusions than the standard transpapillary method [9]. However, there are reports of stent migration into the bile duct, pancreas, or duodenal wall; it seems logical that proximal migrations of stents should occur more frequently with SPS placement [10,11,12].

This meta-analysis aims to identify all available publications investigating patients with biliary strictures of any etiology treated using stent insertion via endoscopic retrograde cholangiopancreatography (ERCP). We aimed to analyze the stent patency and procedure-related complications of the suprapapillary and transpapillary stent positions.

## 2. Materials and Methods

We performed our systematic review and meta-analysis based on the Preferred Reporting Items for Systematic Reviews and Meta-Analyses (PRISMA) Statement [13]. The review protocol was submitted on the 4th of July 2017 in the PROSPERO database (see http://www.crd.york.ac.uk/prospero, accessed on 3 May 2022) as “Suprapapillary versus TPS placement for the management of biliary stenosis—a systematic review and meta-analysis” under the registration number CRD42017069840. Contrary to the protocol, we excluded patients undergoing percutaneous transhepatic cholangiography and used the Risk Of Bias In Non-randomized Studies of Interventions (ROBINS-I) tool to study quality assessment instead of the Methodological Index for Non-Randomized Studies (MINORS) criteria.

### 2.1. Search Strategy and Eligibility Criteria

A comprehensive literature search was conducted up until 20 December 2020 in the electronic databases of the Cochrane Central Register of Controlled Trials (CENTRAL), Embase, and MEDLINE (via PubMed). The synonyms used for suprapapillary stent placement were included in the search strategy. The search terms were: (“intraductal” OR “Oddi sphincter” OR “suprapapillary” OR “inside”) AND “stent”. No restrictions were applied regarding the year of publication or language of the article, and no filters were used. All fields were searched in all databases. The gray literature was excluded except for conference abstracts.

Study eligibility was determined based on our predefined PICO framework. Publications investigating biliary stent placement via ERCP in adult patients with benign or malignant biliary obstruction (P) were included. The stent position had to be transpapillary (I) or suprapapillary (C) and was compared by the following outcomes (O): stent patency time (days), migration rate, cholangitis, pancreatitis, cholecystitis and other procedure-related complications (bleeding or perforation). Definitions of the investigated endpoints were accepted as they appeared in the included individual publications. Regarding study design, randomized controlled trials (RCTs) and prospective or retrospective observational studies were considered eligible if they met the criteria stated in the predefined PICO framework.

We excluded studies that investigated patients with percutaneous stent placement. Research protocols, conference abstracts, and publications without a control group or that did not report any of the investigated endpoints were also excluded.

### 2.2. Selection Strategy and Data Extraction

We used EndNote X9 citation management software (Clarivate Analytics, Philadelphia, PA, USA) to screen all the yielded results. After removing the duplicates, two independent reviewers (D.P. and N.K.) assessed the study eligibility based on titles, abstracts, and full texts. Any disagreements were resolved by consensus. After each selection step, Cohen’s kappa coefficient (ⲕ) was used to measure the inter-rater reliability.

A standardized data collection form was created using Excel software (Office 365, Microsoft, Redmond, WA, USA) to perform data extraction. Data were extracted by two independent review authors (D.P. and N.K.); any disagreements were resolved by consensus. The following data were extracted from each included publication: first author, study design, publication date, study duration, study site, number of centers, inclusion criteria, indication for stent placement, exclusion criteria, biliary stent type, number of patients, age, gender, number of patients in each investigated group, number of events in each examined group concerning the investigated dichotomous endpoints, means, standard deviations, medians, ranges, and interquartile ranges (IQRs) for continuous endpoints.

### 2.3. Risk of Bias Assessment and Certainty of Evidence

Two independent review authors (D.P. and N.K.) assessed the risk of bias to define the quality of the included publications, and disagreements were resolved by consensus. The risk of bias in non-randomized studies was assessed using ROBINS-I (Risk Of Bias In Non-randomized Studies of Interventions) [14]. For RCTs, we used the RoB2 tool recommended by the Cochrane collaboration [15]. The robvis (see https://mcguinlu.shinyapps.io/robvis/, accessed on 15 February 2022) web app tool was used to visualize the risk of bias assessment [16].

Two independent investigators (D.P. and N.K.) used the Grades of Recommendation, Assessment, Development, and Evaluation (GRADE) workgroup recommendations to evaluate the certainty of evidence [17]. Disagreements were resolved by consensus. We built three ‘Summary of findings’ tables, applying the GRADEpro GDT software [18] for each investigated outcome.

### 2.4. Statistical Analysis

All the analyses were performed using R environment (R Core Team (2021), R: A language and environment for statistical computing. R Foundation for Statistical Computing, Vienna, Austria, R version 4.1.2 (1 November 2021)).

For dichotomous outcomes, odds ratios [1], and for continuous variables, weighted mean differences (WMDs) were calculated with 95% confidence intervals (CIs). In the cases of missing mean values or standard deviations, we used Wan’s method [19] or followed the suggestion of the Cochrane Handbook [19], respectively.

A p-value less than 0.05 was considered a statistically significant difference. We used the random effects model to calculate the overall estimates using the DerSimonian–Laird [20] method. The results of the meta-analyses are presented in forest plots. We used a random effects model to calculate the overall estimates using the restricted maximum likelihood estimator [21] to calculate the heterogeneity variance τ 2.

Heterogeneity was tested using the I^2^ statistics. Following the Cochrane Handbook [19], I^2^ represents the magnitude of the heterogeneity (‘Might not be important’: 0–40%, ‘Moderate’: 30–60%, ‘Substantial’: 50–90% and ‘Considerable’: 75–100%). Heterogeneity with a *p*-value < 0.1 is considered significant.

Egger’s tests and funnel plots were used to assess any publication bias for the outcomes having at least 10 studies included. If at least six studies were available, we only performed funnel plots.

## 3. Results

### 3.1. Systematic Search and Selection

**Figure 1:**
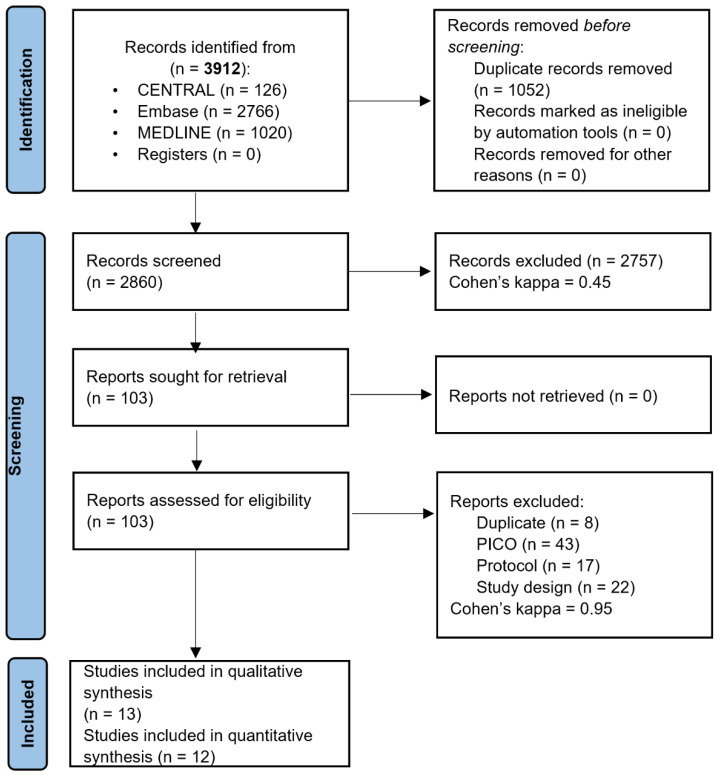
PRISMA 2020 flow diagram.

Our systematic search identified 13 eligible publications out of 3912 records. Thirteen articles were eligible [3,8,12,22,23,24,25,26,27,28,29,30,31] for qualitative synthesis, and twelve were included in the quantitative synthesis [3,8,12,22,23,24,25,26,27,28,29,31]. The reasons for excluding specific publications on the full-text level are presented in Supplementary Table S1. The study search and selection are summarized in the PRISMA flow diagram (Figure 1).

### 3.2. Description of the Selected Studies

Three prospective [8,12,28] and ten retrospective studies [3,22,23,24,25,26,27,29,30,31] were identified during the literature search. Only two studies were RCTs [12,28]. Four of the included publications were abstracts [28,29,30,31]. The eligible articles were published between 1992 and 2019, mainly in Asia [3,8,24,25,26,27,28,30]. Two studies were published in North America [22,23] and one in Europe [12]. Two of the studies were multicentric [23,28], while the others were single-center studies [3,8,12,22,24,25,26,27,30], or data were not available regarding this information [29,31]. In 12 publications, only malignant etiologies were analyzed [3,8,12,23,24,25,26,27,28,29,30,31] and in one study, both benign and malignant etiologies of biliary obstruction were analyzed [22]. The characteristics of the included studies are presented in Table 1.

### 3.3. Stent Patency

**Figure 2:**
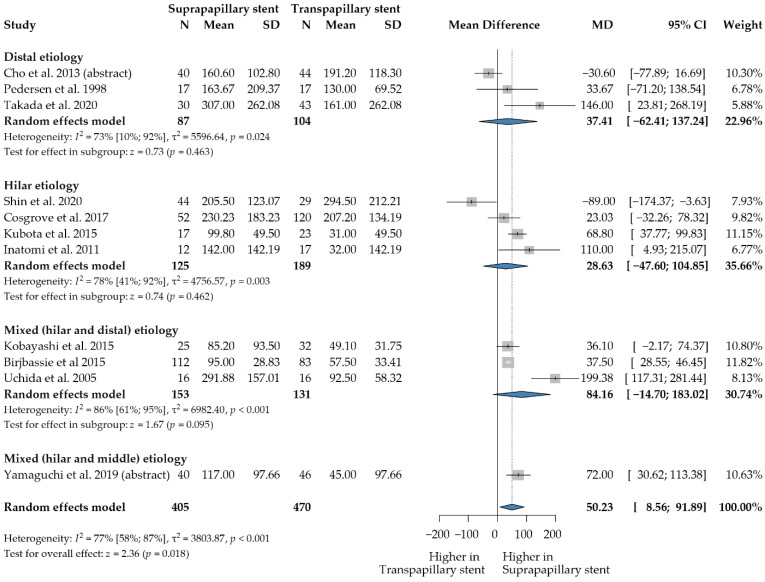
Forest plot comparing the stent patency time between suprapapillary and transpapillary stents. Unit of measurement: days. WMD: weighted mean difference; *p*: *p*-value; CI: confidence interval; I-squared: I^2^. [3,8,12,22,23,24,25,26,27,28,31].

Stent patency time was analyzed in 11 studies involving 875 patients [3,8,12,22,23,24,25,26,27,28,31]. Stent patency time was significantly longer in the SPS group (WMD = 50.23 days, 95% CI: 8.56; 91.89; *p* = 0.018; heterogeneity: I^2^ = 77%, *p* < 0.001) (Figure 2). A similar result was obtained after only analyzing the full texts reporting on malignant indications (WMD = 62.30 days, 95% CI: 4.39, 120.21; *p* = 0.035; heterogeneity: I^2^ = 76.0%, *p* < 0.001) [3,8,12,23,24,25,26,27] (Supplementary Figure S1).

We analyzed the patency times for SPS and TPS positions separately for metal and plastic stents. Self-expanding metal stents were used in five studies involving 597 patients [3,22,23,27,28]. No significant difference was found between the SPS and TPS positions (WMD = 10.85 days, 95% CI: −48.23, 69.94; *p* = 0.719; heterogeneity: I^2^ = 79%*, p <* 0.001) for metal stents (Supplementary Figure S2). Only investigating malignant indication showed similar results; no significant differences were found (WMD = 3.98 95% CI: −79.63; 87.59; *p* = 0.926; heterogeneity I^2^ = 74%*, p =* 0.009) [3,23,27,28].

Six publications with 278 patients were pooled in the plastic stent subgroup [8,12,24,25,26,31]. Plastic stents in the SPS position had a longer stent patency time (WMD = 80.49 days, 95% CI: 37.57, 123.40, *p* < 0.001; heterogeneity: I^2^ = 63%*, p =* 0.019) (Supplementary Figure S3).

### 3.4. Stent Migration

**Figure 3:**
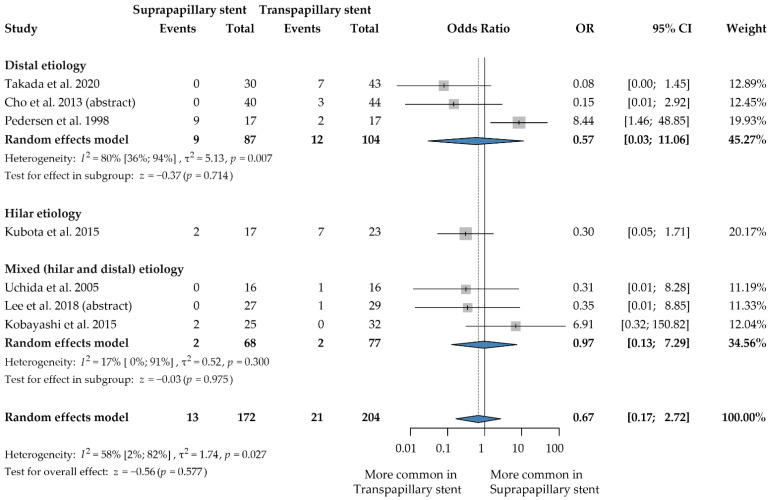
Forest plot comparing stent migration rate between suprapapillary and transpapillary stents. OR: odds ratio, p: p-value; CI: confidence interval; I-squared: I^2^. [3,8,12,25,26,28,29].

Our meta-analysis, including seven articles with 376 patients [3,8,12,25,26,28,29], found no difference concerning stent migration between the two techniques (OR: 0.67, 95% CI: 0.17, 2.72; *p* = 0.577; heterogeneity: I^2^ = 58%*, p =* 0.027) (Figure 3). Only one trial found significantly more stent migration in the SPS placement compared to TPS [12]. All the other studies reported no significant differences in this regard.

The subgroup analysis of plastic stent placement, investigating four publications involving 163 patients [8,12,25,26], did not find any difference between the two techniques (OR: 1.57, 95% CI: 0.25, 9.83; *p* = 0.627; heterogeneity: I^2^ = 66%, *p* = 0.032) (Supplementary Figure S4).

### 3.5. Cholangitis

**Figure 4:**
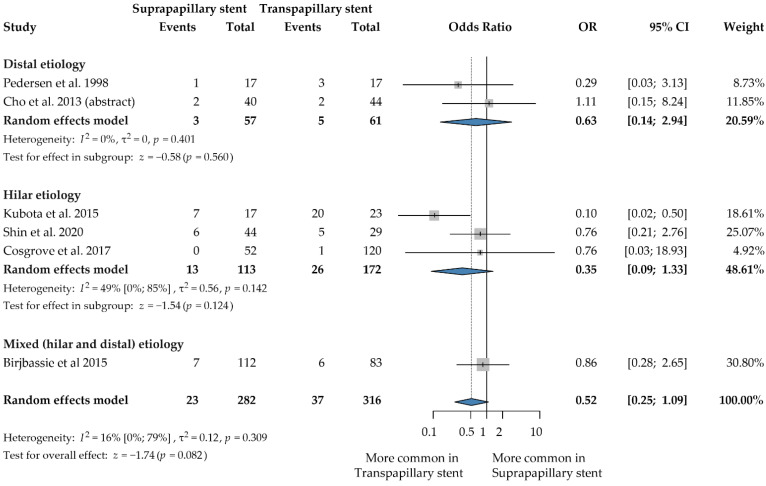
Forest plot comparing cholangitis rate between suprapapillary and transpapillary stents. OR: odds ratio, *p*: *p*-value; CI: confidence interval; I-squared: I^2^. [12,22,23,26,27,28].

Six studies with a total of 598 patients contained data on cholangitis rates [12,22,23,26,27,28]. Only one study found that the SPS placement causes significantly less cholangitis than the transpapillary method [26]. All the other articles reported similar rates of this complication in the two groups. The rate of cholangitis was similar across the two investigated groups (OR: 0.52, 95% CI: 0.25, 1.09; *p* = 0.082; heterogeneity: I^2^ = 16%, *p* = 0.309) (Figure 4). There is a clearly visible tendency toward lower cholangitis rates with the suprapapillary position.

On the other hand, there was a significantly lower risk of cholangitis with SPS when we analyzed full texts only including a malignant indication (OR: 0.34, 95% CI: 0.13, 0.93); *p* = 0.036; heterogeneity: I^2^ = 24%, *p* = 0.269) (Supplementary Figure S5) [12,23,26,27]. 

There was no difference in cholangitis between SPS and TPS in the subgroup of metal stents (OR: 0.85, 95% CI: 0.40, 1.81; *p* = 0.665; heterogeneity: I^2^ = 0.0%, *p* = 0.992) (Supplementary Figure S6) [22,23,27,28]. Only investigating malignant indication showed similar results; no significant difference was found (OR = 0.84 95% CI: 0.30; 2.34; *p* = 0.753; heterogeneity I^2^ = %*, p =* 0.951) [23,27,28]. 

### 3.6. Pancreatitis

**Figure 5:**
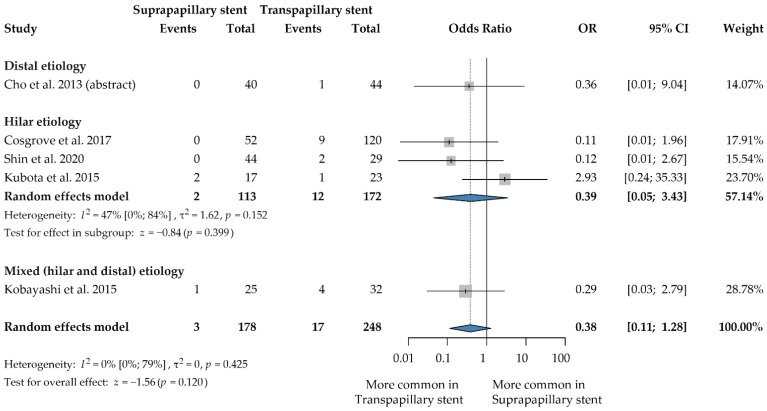
Forest plot comparing pancreatitis rate between suprapapillary and transpapillary stents. OR: odds ratio, p: P-value; CI: confidence interval; I-squared: I^2^. [23,25,26,27,28].

Five articles including a total of 426 patients had data on the pancreatitis rate [23,25,26,27,28]. The results of the meta-analysis showed a similar rate of pancreatitis across the groups (OR: 0.38, 95% CI: 0.11, 1.28; *p* = 0.120; heterogeneity: I^2^ = 0.0%, *p* = 0.425) (Figure 5).

After a sensitivity analysis leaving out the study only reported as an abstract [28], we found the same result (OR: 0.38, 95% CI: 0.08, 1.66; *p* = 0.197; heterogeneity: I^2^ = 22%, *p* = 0.277) (Supplementary Figure S7). In the metal stent subgroup, the suprapapillary method had a significantly lower rate of pancreatitis (OR: 0.16, 95% CI: 0.03, 0.95; *p* = 0.043; heterogeneity: I^2^ = 0.0%, *p* = 0.850) (Supplementary Figure S8) [23,27,28]. 

### 3.7. Cholecystitis

Three articles incorporating a total of 230 patients investigated cholecystitis in the case of metal stents [3,27,28]; our analysis showed similar rates of cholecystitis in the two groups (OR: 1.41, 95% CI: 0.28, 7.15; *p* = 0.677; heterogeneity: I^2^ = 0%, *p* = 0.455) (Supplementary Figure S9).

### 3.8. Other Complications

Seven studies [8,12,23,24,25,28,31] reported the rates of bleeding complications, but it was a rare complication that was mostly the consequence of endoscopic sphincterotomy [32]. Only one bleeding complication in the suprapapillary [25] and two in the transpapillary group [23] were reported. Only three perforations were reported, two in the transpapillary and one in the SPS placement group [23]. We summarize the procedure-related complications, EST, and survival rates in Supplementary Table S2.

### 3.9. Risk of Bias Assessment

We assessed the quality of each included publication used for quantitative synthesis. The study- and domain-level results for each outcome are detailed in the supplementary material (Supplementary Figures S10–S27). In the included non-randomized publications [3,8,22,23,24,25,26,27,29,30,31], the risks of bias at pre-, at-, and post-intervention levels were judged to be at low risk of bias, except for the domains of “bias due to confounding” and “bias in selection of reported results”, where we judged serious and moderate risk in most of the studies, respectively. The overall risk of bias was mainly judged as a serious risk. For the two included randomized trials [12,28], we judged “some concerns” at the “randomization process” domain in one study [28], and we also had some concerns in the “selection of the reported result” domain in both cases. The overall risk of bias was judged to be at “some concerns”.

### 3.10. Publication Bias

In the case of stent patency time, we performed the Egger’s test and a funnel plot. No publication bias was detected (*p* = 0.591). We performed funnel plots for the following outcomes: patency (subgroup plastic stents), stent migration, and cholangitis. Based on visual inspection, publication bias was suspected in the case of stent migration and cholangitis. The funnel plot of each outcome can be found in the supplementary material (Supplementary Figures S28–S32).

### 3.11. Certainty of Evidence

The investigated outcomes were assessed as having a low to very low level of evidence. The design of the included studies, the presence of serious risk of bias, the possible inconsistency due to heterogeneity, and the serious risk of imprecision greatly influenced the quality of evidence. The summary of findings tables are shown in Supplementary Tables S3–S6. The first table comprises stent patency, stent migration, post-ERCP cholangitis, and pancreatitis (Supplementary Table S3); the second and third tables include the level of evidence for the subgroups of metal and plastics stents (Supplementary Tables S4 and S5), respectively. The last table (Supplementary Table S6) consists of the level of evidence for the studies published as full-text articles.

## 4. Discussion

This meta-analysis revealed that the suprapapillary positioning of stents in biliary obstruction is beneficial. We found significantly longer patency times in all cases. Lower rates of cholangitis and pancreatitis were found in the subgroups of full texts investigating only malignant indications and metal stents, respectively. Furthermore, the stent migration rates were equal in the investigated groups.

### 4.1. Stent Patency

Longer stent patency is an interest to both patients and physicians. There have been multiple attempts to prevent or delay stent occlusion: covering stents to hinder tumor ingrowth [33] and biofilm formation [34], developing an anti-reflux valve against duodenobiliary reflux, [35] and experiments with larger stent diameters [4]. Extensive research has been undertaken to examine the effect of the position of suprapapillary stents on stent function time. A previous review attempted to explain why it might result in longer patency [9]. It mentions that this position might prevent sludge formation by hindering food impaction and biofilm formation; it might also mitigate duodenobiliary reflux by preserving the SO as a natural mechanical barrier [22].

We found significantly longer stent patency times in the suprapapillary group. Six pooled publications favored SPS; in contrast, only one article found a longer patency time in TPS. The authors explain this by the higher stent migration rate of the suprapapillary group [12]. They also suggested that SPS might be recommended if the high migration rate could be resolved.

In the case of the metal stent subgroup, there was no significant difference in the patency time between the two positions. A previous publication assumed that the advantage of SPS cannot be assessed in the case of metal stents since the stent material significantly reduces the occlusion from debris; thus, the effect of stent position might be neutralized [23].

There are advantages of metal stents over plastic ones; for instance, SEMSs provide longer patency in the transpapillary position [36]. On the other hand, they are more expensive, [37] their placement frequently requires EST, and their removal is more difficult [24]. Plastic stents are substantially less expensive, and it is easier to handle them in surgical situations; however, they occlude faster [36]. Consequently, their replacement is more common.

To slow down the process of occlusion, the suprapapillary position might be a solution. A major concern against SPS is that stent removal can be problematic [12]. To ease the removal of SPS, a nylon thread attached to the stent could be a solution, as some authors employed in their work. [24,25,26] Inatomi et al. found that threaded SPS plastic stents without EST had significantly longer patency times compared to metal stents in the suprapapillary position [24].

The rate of pancreatic cancer among the included patients likely influences the results of SPS. Matsushita et al. suggest that SPS insertion is ineffective in the case of pancreatic cancer [38]. The distance from the ampulla and the biliary obstruction affect stent patency as well [9]. Presumably, EST influences the effectiveness of SPS. Takada et al. found that SPS without EST has longer patency than with EST [3]. This might be because EST breaches the barrier function of the SO and consequently causes duodenobiliary reflux and stent occlusion. The patency time did not differ between EST and no EST in the TPS group. Kuwatani et al. suggest EST should be avoided in the case of SPS [9].

### 4.2. Stent Migration

The higher migration rate was a major concern regarding SPS [12]. According to one of the earliest and most highly cited articles, stent migration is more frequent in the case of SPS, and the authors recommend using TPS [12]. However, they cut off the distal flap of the stents in half of the patients, and most patients had pancreatic cancer (28/34), which can cause severe axis deviation, promoting stent migration [38]. Additionally, Uchida et al. comment that the Amsterdam stent type is not suitable due to its stiffness, and a more flexible Tannenbaum stent should be used to prevent stent migration [8]. We found a surprising trend resulting in a lower rate of SPS migration appearing in most of the investigated studies; the results are not significant, however.

One study that tried to decrease stent migration with Tannenbaum stents found a lower rate of SPS migration; however, it was not significant [8]. A Tannenbaum stent is flexed at the central part, and four additional radial flaps are placed at both ends of the stent. Kubota et al. assumed that because the two anatomical sites, the ampulla and tumor lesion, where TPS stents are attached, can move independently, this may facilitate TPS migration [26]. In the case of uncovered SEMS, migration occurs less frequently than in covered [39] or plastic stents.

### 4.3. Cholangitis

Previous studies cite that TPS may lead to reflux cholangitis [40]. Stent placement above the SO has been proposed to decrease rates of complications by allowing an intact SO to act as a physiological barrier against the reflux of bacteria and debris into the common bile duct [23]. The development of cholangitis might result from a combination of digestive juice reflux and the incomplete drainage of the biliary tract [26]. We did not find a significant difference; however, a tendency observed in the SPS position might result in a lower overall rate of cholangitis. We found a significantly lower rate of cholangitis by only analyzing full-text articles, including only malignant indications. In the subgroup only investigating metal stents, we found no significant difference. In a previous study, Okamoto et al. found a positive association between transpapillary CSEMS and cholangitis [40]. EST with suprapapillary SEMS insertion is linked to a higher rate of cholangitis [41].

### 4.4. Pancreatitis

Pancreatitis is a severe complication following ERCP. Theoretically, the SPS position might lower the rate of post-procedural pancreatitis since it may decrease the burden on the major duodenal papilla [9]; thus, it does not obstruct the secretion of pancreatic juice into the duodenum [42]. We found no significant difference between TPS and SPS; however, a trend for a lower rate of pancreatitis in the SPS group also appeared. A significantly lower rate of pancreatitis occurred in the metal stent subgroup. A previous study found a lower rate of pancreatitis in the SPS group [23]. The authors assume that two TPS might lead to pancreatic outflow obstruction by mechanical obstruction. Placing a SEMS significantly increases the rate of post-ERCP pancreatitis compared to plastic stents [43], and larger-diameter stents might increase the risk of developing pancreatitis [26].

EST is used to prevent post-ERCP pancreatitis in TPS placement by reducing the burden on the pancreatic duct [44]. However, a previous meta-analysis found no such effect of EST in the prevention of pancreatitis [42]. 

### 4.5. Strengths and Limitations

This is the first and most comprehensive meta-analysis and systematic review that synthesizes the available information regarding suprapapillary and transpapillary biliary stent insertion via ERCP. Our methods are transparent and reproducible; we used a strict methodology during the research.

Our study has limitations. Most of the included studies were non-randomized, non-prospective publications; therefore, they provide low-quality data and evidence, and studies published only in abstract form were also included. The presence of confounding factors in the included studies is likely, and they were mainly judged as carrying a serious risk of bias. The populations of the included studies have heterogeneous etiology of biliary obstruction. In some studies, patients received additional and/or adjunctive therapy and not just palliative treatment for biliary obstruction. Some authors used total or partial EST, not just for transpapillary placement but also for SPS insertion, influencing the SOD’s natural protective effect on duodenobiliary reflux. In the case of stent patency time and migration, the pooled publications showed substantial heterogeneity. Additionally, SPS can only be applied for patients with intact lower bile ducts, because obstruction must be sufficiently distant from the ampulla to perform such insertion. Pancreatic cancers cause distal bile duct obstruction, in most cases in close proximity to the papilla; therefore, in this type of bile duct obstruction, suprapapillary stent placement is usually not feasible. We would not advise SPS in pancreatic cancer even if there is a sufficient distance between the obstruction and the papilla initially, because during the progression of the tumor, the distance becomes shorter. Moreover, some cases, hilar strictures demand multiple SPS stent insertions into the bile ducts, which could have influenced our results. Publication bias could not be assessed in every case due to the low number of studies.

### 4.6. Implication for Practice

Currently, there is no clear evidence on which stent position should be used in biliary obstructions. According to our findings, the suprapapillary position could be an alternative to the more commonly used transpapillary position. Suprapillary stenting is associated with longer stent patency and fewer complications in some cases, but with a similar migration rate. These advantages might result in fewer additional interventions; thus, the patients’ quality of life might improve, and healthcare costs would be lower. Regarding stent revision, threads have been added to the distal ends of plastic and metal stents as well; this approach might ease the removal of the stents.

### 4.7. Implications for Research

The possible beneficial effect of the SPS position in biliary obstruction can be seen. Unfortunately, only a few RCTs are available; therefore, further RCTs are needed to prove the beneficial effect of SPS positioning in biliary obstructions. These trials should investigate the feasibility and effect of suprapapillary stenting in benign and malignant etiologies. The main features of a stent (material, size, length, etc.) are likely important as well. It might be advisable for future RCTs to address questions about stent insertion success rate, endoscopic revision success rate, stent removability or retrieval, stent patency time, and post-procedural complication rates of the two stent positions. Cost-effectiveness analyses might also be helpful for future guidelines. The placement of multiple stents could be an additional option to optimize stent patency further. A possible trial for optimizing indwelling stents’ performance could be comparing suprapapillary threaded PS and MS.

## 5. Conclusions

Based on our results, suprapapillary stenting has an advantage over the TPS placement in some types of malignant biliary obstructions, as it could result in longer stent patency times. We could not find a difference in the stent migration rate between the two stent placement methods, making suprapapillary stenting safe. However, the low number and suboptimal quality of the studies call for more randomized controlled trials to find the proper indication of SPS positioning.

## Figures and Tables

**Table 1:** Characteristics of included studies.

Author, Year, Country, Number of Centers	Study Design	Time of Enrollment	N^0^ of Patients (Age, N^0^ of Females)	Indication(s) for Stent Placement	Exclusion Criteria	Stent Type (TPS vs. SPS)	EST (TPS, SPS)	Outcome(s)
Brijbassie et al. 2015 [2], USA, 1 center	Retrospective case series	2006–2009	195 patients (mean age 67.1 ± 12.2 years, 75)	Benign and malignant biliary strictures	N/A	metal (FCSEMS) vs. metal (FCSEMS)	yes, partial	Stent patency, post-ERCP cholangitis
Cho et al. 2013 [3] (abstract), Japan, 6 centers	Prospective, randomized trial	2010–2012	84 patients (mean age 72 ± 12.5, N/A)	Unresectable malignant biliary obstruction	Ampullary cancer, Klatskin tumor, combined intrahepatic bile duct cancer, hemobilia, previous history of biliary drainage	metal (CSEMS) vs. metal (CSEMS)	yes, no	Stent patency, stent migration, post-ERCP cholangitis, post-ERCP pancreatitis, other procedure-related complications: bleeding or cholecystitis
Cosgrove et al. 2017 [4], USA, 3 centers	Retrospective cohort	2007–2013	172 patients (mean age 66.5 ± 14.18, 66)	Unresectable malignant hilar biliary strictures of any etiology	Curative surgical resection	bilateral metal (SEMS) vs. bilateral metal (SEMS)	yes, based on the endoscopist decision (108, 5)	Stent patency, stent migration, post-ERCP cholangitis, post-ERCP pancreatitis, other procedure-related complications: bleeding or perforation
Inatomi et al. 2011 [5], Japan, 1 center	Retrospective cohort	2007–2011	42 patients (67.5 ± 12.2 years, 20)	Unresectable malignant hilar biliary obstruction	N/A	plastic + metal (uncovered) vs. plastic (threaded)	only in metal stents	Stent patency, stent migration, other procedure-related complications: bleeding or perforation
Kobayashi et al. 2015 [6], Japan, 1 center	Retrospective cohort	2006–2011	57 patients (median age 71 (56–86), 12)	Primary biliary duct cancer	Percutaneous transhepatic biliary, pancreatic cancer, ampullary cancer, and intra hepatic cancer	plastic vs. plastic	yes (3,3)	Stent patency, stent occlusion, stent migration, post-ERCP cholangitis, post-ERCP pancreatitis, other procedure-related complications: bleeding, biliary and pancreatic fistula, or liver abscess
Kubota et al. 2015 [7], Japan, 1 center	Retrospective cohort	2012–2015	40 patients (mean age 70, 13)	Primary biliary duct cancer	Gallbladder cancer, intrahepatic cholangiocarcinoma, and biliary cancer arising from pancreatic head lesions and metastatic Klatskin tumors	plastic vs. threaded plastic	yes (multiple TPS)	Stent patency, stent occlusion, stent migration, post-ERCP cholangitis, post-ERCP pancreatitis
Lee et al. 2018 (abstract) [8], N/A	Retrospective cohort	2015–2017	56 (N/A, N/A)	obstructive jaundice due to resectable extrahepatic malignant biliary obstruction	N/A	plastic vs. metal (FCSEMS)	N/A	Stent occlusion, stent migration
Pedersen et al. 1998 [9], Denmark, 1 center	Prospective, randomized trial	1992–1996	34 patients (median age 73.5 (IQR: 67–80), 21)	Malignant biliary obstruction	Curable lesion was suspected or if liver metastases were present at the time of the ERCP	plastic vs. plastic	No	Stent patency, stent occlusion, stent migration, post-ERCP cholangitis, post-ERCP pancreatitis, other procedure-related complications: bleeding, cholecystitis, or perforation
Shin et al. 2020 [10], Korea and Japan, 1 center	Retrospective cohort	2005–2015	73 patients (median age 75 (49–90), 36)	Hilar cholangiocarcinoma	Previous history of SEMS treatment, CCC involving the duodenum, prior history of gastrointestinal surgery, failure of endoscopic SEMS insertion, or SEMS insertion by a modality other than endoscopy	metal (SEMS) vs. metal (SEMS)	Yes (all)	Stent patency, stent occlusion, post-ERCP cholangitis, post-ERCP pancreatitis, procedure-related complications: cholecystitis
Takada et al. 2020 [11], Japan, 1 center	Retrospective cohort	2014–2016	73 patients (median age 69 (52–86), 38)	Unresectable distal malignant biliary obstruction	Duodenal stents or they underwent concurrent placement of plastic stents and SEMS	metal (SEMS: covered+ uncovered) vs. metal (SEMS: covered + uncovered)	yes (12,10)	Stent patency, stent occlusion, stent migration, other procedure-related complications: cholecystitis, liver abscess, or liver hematoma
Taniguchi et al. 2020 (abstract) [12], Japan, 1 center	Retrospective cohort	2016–2019	96 patients (N/A, N/A)	Nonhilar, extrahepatic, malignant biliary stricture	N/A	metal (covered) vs. metal (covered)	N/A	Stent patency, stent occlusion
Uchida et al. 2005 [13], Japan, 1 center	Prospective, non-randomized	1999–2003	32 patients (mean age 75 (56–92), 15)	Unresectable and previously untreated malignant biliary obstruction	Lesions involved the bifurcation of the common hepatic bile duct; distance from the stenosis to the sphincter of Oddi was less than 15 mm on X-ray examination	plastic vs. plastic	No	Stent patency, stent occlusion, stent migration, post-ERCP pancreatitis, post-ERCP cholangitis, other procedure-related complications: bleeding, cholecystitis, or perforation
Yamaguchi et al. 2019 (abstract) [14], N/A	Retrospective cohort	2008–2018	74 patients (N/A, N/A)	Unresectable malignant hilar or middle bile duct obstruction	N/A	plastic vs. plastic	N/A	Stent patency, stent migration, post-ERCP cholangitis, post-ERCP pancreatitis, other procedure-related complications: bleeding or cholecystitis

TPS: transpapillary stent, SPS: suprapapillary stent, EST: endoscopic sphincterotomy, SEMS: self-expandable metallic stent, ERCP: endoscopic retrograde cholangio-pancreatography, N/A: not available.

## Data Availability

No new data were created or analyzed in this study. Data sharing is not applicable to this article.

## Supplementary Materials

The following supporting information can be downloaded at: https://www.mdpi.com/article/10.3390/jcm12030898/s1.

## Abbreviations

CI	Confidence interval
ERCP	Endoscopic retrograde cholangiopancreatography
IQR	Interquartile ranges
OR	Odds ratio
RCT	Randomized controlled trial
SO	Sphincter of Oddi
SPS	Suprapapillary stent
SEMS	Self-expandable metallic stent
TPS	Transpapillary stent
WMD	Weighted mean difference
