# Supplementary material for: Suprapapillary Biliary Stents Have Longer Patency Times than Transpapillary Stents—A Systematic Review and Meta-Analysis
Source: J Clin Med. 2023 Jan 23;12(3):898. doi: 10.3390/jcm12030898 (PMC9917857; doi:10.3390/jcm12030898)
Supplement: Supplementary file 1 [file jcm-12-00898-s001.zip › jcm-2142660-supplementary.pdf]

Journal of Clinical Medicine

Supplementary Material

Tables

Table S1. List of excluded publications on the full-text level.

	Study	Reason for exclusion
Aepli et al. 2017 [1]	Success and complications of an intra-ductal fully covered self-expanding metal stent (ID-FCSEMS) to treat anastomotic biliary strictures (AS) after orthotopic liver transplantation (OLT)	PICO
Al-Kawas et al. 2005 [2]	Biliary access during endoscopic retrograde cholangiopancreatography: How to precut and a word of caution!	Study design
Aljahdli et al. 2018 [3]	Management of distal malignant biliary obstruction	Study design
Barresi et al. 2016 [4]	A Challenging Diagnosis for a Biliary Stricture	Study design
Bhalla et al. 2019 [5]	Endoscopic Management of Acute Cholecystitis Following Metal Stent Placement for Malignant Biliary Strictures: A View from the Inside Looking in	Study design
Brijbassie et al. 2015 [6]	Transpapillary vs. intraductal fully covered selfexpanding metal stent placement for malignant and benign biliary disease: Does it make a difference?	Duplicate
Chang et al. 2010 [7]	Usefulness of the rendezvous technique for biliary stricture after adult right-lobe living-donor liver transplantation with duct-to-duct anastomosis	PICO
Chang et al. 2012 [8]	Comparative study of rendezvous techniques in post-liver transplant biliary stricture	PICO
Chantarojanasiri et al. 2018 [9]	Usefulness of stent placement above the papilla, so-called, 'inside stent'	Study design
ChiCtr. 2019 [10]	Comparative study for the efficacy of intraductal RFA + biliary stent placement or intraductal Iodine-125 seed + biliary stent placement by percutaneous transhepatic cholangial drainage for treating the patients with unresectable malignant biliary strictures	Protocol
ChiCtr. 2019 [11]	Comparative study of the efficacy of intraductal RFA +stent placement by ERCP and percutaneous transhepatic approach in patients with unresectable malignant biliary strictures	Protocol
Choi et al. 2015 [12]	Feasibility of self expandable metal stent for preservation of sphincter of Oddi function in patients with common bile duct stones a pilot study	PICO
Choi et al. 2019 [13]	Newly modified plastic stent for transhepatic placement above the sphincter of Oddi in treatment of biliary anastomotic stricture after liver transplantation: Preliminary report of 7 patient	PICO
Choi et al. 2020 [14]	Abstract No. 579 Newly modified plastic stent for transhepatic placement above the sphincter of Oddi in treatment of biliary anastomotic stricture after liver transplantation: preliminary report of seven patients	PICO
Dawwas et al. 2016 [15]	Endoscopic assessment and management of biliary strictures	Study design
Douhara et al. 2016 [16]	Inside plastic stent is useful and safety for malignant biliary stenosis including Bismuth IV	PICO

García et al. 2013 [17]	Difficulties encountered for removing fully covered self-expanding metal stents inserted in benign biliary conditions	PICO
Gargouri et al. 2010 [18]	[Biliary stent occlusion]	PICO
Hasegawa et al. 2014 [19]	Appropriate therapeutic strategy for biliary stenting using threaded inside stents based on the location of the malignant obstruction	PICO
Hatzidakis et al. 2001 [20]	Nitinol stents for palliative treatment of malignant obstructive jaundice: Should we stent the sphincter of Oddi in every case?	PICO
Hayasaka et al. 2014 [21]	Endoscopic placement of a large-bore covered self-expandable metallic stent for cholangitis caused by mucus from a pancreatic mucinous neoplasm	Study design
Hayashi et al. 2018 [22]	Endoscopic management of unresectable malignant hilar strictures using threaded inside stents versus metallic stents	PICO
Hyodo et al. 1998 [23]	Endoscopic metallic stenting and intraductal ultrasonography in a case of bile duct cancer	Study design
Inatomi et al. 2011 [24]	The efficacy and safety of threaded biliary plastic "inside-stent" for malignant hilar obstruction	Duplicate
Inoue et al. 2020 [25]	Double radiofrequency ablation with metal stent placement for refractory benign biliary and pancreatic duct strictures	Study design
Inoue et al. 2020 [26]	Intraductal placement of a fully covered metal stent with a long string for distal malignant biliary obstruction without endoscopic sphincterotomy: Prospective multi-center feasibility study	PICO
Ishiwatari et al. 2013 [27]	Newly designed plastic stent for endoscopic placement above the sphincter of Oddi in patients with malignant hilar biliary obstruction	PICO
Itoi et al. 2013 [28]	Current status and issues regarding biliary stenting in unresectable biliary obstruction	Study design
Jo et al. 2015 [29]	Suprapapillary versus transpapillary stent placement for malignant biliary obstruction: which is better?	PICO
Jo et al. 2014 [30]	Supra-papillary versus trans-papillary biliary stent for malignant biliary obstruction: Which is the better?	Conference asbtract
Jprn, Umin. 2014 [31]	Efficacy and Safety of inside stent for preoperative biliary drainage in patients with malignant hilar biliary obstruction: prospective,multicenter,single-arm study	Protocol
Jprn, Umin. 2014 [32]	Efficacy and safety of inside stent in patients with biliary stricture: a prospective multicenter study	Protocol
Jprn, Umin. 2016 [33]	The efficacy and safety of non-flared fully covered self expandable metalic stent for benign biliary duct stricture	Protocol
Jprn, Umin. 2016 [34]	Usefulness of Non-flared Intraductal Fully Covered Metallic Stent for Refractory Biliary Strictures after Living Donor Liver Transplantation	Protocol
Jprn, Umin. 2014 [35]	A Newly Modified Non-Flared Fully Covered Metallic Stent of 12 mm-Diameter with Long Lasso for Intraductal Placement in Patients with Malignant Biliary Stricture: feasibility Study	Protocol
Jprn, Umin. 2011 [36]	Plastic stent placement above the sphincter of Oddi ("inside-stent") for patients of malignant hilar biliary obstruction. A prospective phase II trial	Protocol
Jprn, Umin. 2011 [37]	Endoscopic treatment of malignant bile duct stricture; Comparative study of conventional stent vs inside stent	Protocol
Jprn, Umin. 2011 [38]	Inside-stent placement for malignant hilar obstruction	Protocol

Jprn, Umin. 2018 [39]	Randomized control trial of inside stenting using plastic stent and side by side stenting using 6mm full-covered self expandable metallic stent for endoscopic bilateral biliary drainage to malignant hilar biliary obstruction	Protocol
Jprn, Umin. 2017 [40]	Randomized controlled trial on efficacy of metal stents vs. inside plastic stents for unresectable malignant obstruction of the perihilar bile duct	Protocol
Jprn, Umin. 2017 [41]	Safety and efficacy of preoperative biliary drainage using plastic stent above the sphincter of Oddi (Inside-stent) in patients with malignant hilar biliary obstruction: a prospective study	Protocol
Jprn, Umin. 2017 [42]	The Usefulness of Newly Modified Non-flared Fully Covered Metal Stent of 12 mm in diameter Comparing with Conventional Stent for Periampullary Malignant Biliary Strictures	Protocol
Jprn, Umin. 2019 [43]	Efficacy of inside plastic stent placement for malignant hilar biliary obstruction -multicenter, randomized control trial	Protocol
Jun et al. 2015 [44]	Feasibility of self-expandable metal stents for preservation of sphincter of Oddi function in patients with common bile duct stones: A pilot study	PICO
Kaneko et al. 2014 [45]	Efficacy of plastic stent placement inside bile ducts for the treatment of unresectable malignant hilar obstruction (with videos)	PICO
Kawakami et al. 2012 [46]	Covered metallic stent for ischemic hilar biliary stricture	Study design
Kogure et al. 2011 [47]	Plastic stent placement above the sphincter of Oddi ("inside stent") for biliary strictures	PICO
Kogure et al. 2012 [48]	Endoscopic management of biliary strictures after adult living donor liver transplantation with duct-to-duct reconstruction	PICO
Kogure et al. 2013 [49]	Endoscopic management of biliary strictures after adult living donor liver transplantation with duct-to-duct reconstruction	PICO
Koizumi et al. 2020 [50]	Endoscopic stent placement above the sphincter of Oddi for biliary strictures after living donor liver transplantation	PICO
Koizumi et al. 2019 [51]	ENDOSCOPIC STENT PLACEMENT ABOVE THE SPHINCTER OF ODDI FOR BILIARY STRICTURES AFTER LIVING DONOR LIVER TRANSPLANTATION	PICO
Kubota et al. 2015 [52]	Inside stent and neoadjuvant chemotherapy can provide a chance of surgery in patients with symptomatic, initially unresectable klatskin tumors	Duplicate
Kurita et al. 2013 [53]	Endoscopic stent placement above the intact sphincter of Oddi for biliary strictures after living donor liver transplantation	PICO
Lee et al. 2019 [54]	Usefulness of newly modified fully covered metallic stent of 12 mm in diameter and anti-migration feature for periampullary malignant biliary strictures: Comparison with conventional standard metal stent	PICO
Lee et al. 2016 [55]	Biliary intraductal metastasis from advanced gastric cancer: radiologic and histologic characteristics, and clinical outcomes of percutaneous metallic stent placement	PICO
Lee et al. 2016 [56]	Newly developed flower-type covered selfexpandable metal stent for preventing cholecystitis and pancreatitis: Physical properties	PICO
Lee et al. 2000 [57]	Percutaneous placement of self-expandable metallic biliary stents in malignant extrahepatic strictures: indications of transpapillary and suprapapillary methods	PICO
Leung et al. 2008 [58]	Comparison of accessory performance using a novel ERCP mechanical	PICO

	simulator	
Li et al. 2014 [59]	Treatment experience of fully covered self-expandable metal stents in biliary stricture after liver transplantation	PICO
Liu et al. 1998 [60]	Feasibility of stent placement above the sphincter of Oddi ("inside-stent") for patients with malignant biliary obstruction	PICO
Moon et al. 2012 [61]	Feasibility of placing a modified fully covered self-expandable metal stent above the papilla to minimize stent-induced bile duct injury in patients with refractory benign biliary strictures (with videos)	PICO
Moon et al. 2015 [62]	A newly modified non-flared fully covered metallic stent of 12 mm-diameter with long lasso for intraductal placement in patients with malignant biliary stricture: Feasibility study	PICO
Moon et al. 2012 [63]	Biliary drainage with a modified fully covered self-expandable metallic stent for potentially resectable distal malignant biliary obstruction	PICO
Moon et al. 2016 [64]	Usefulness of non-flared intraductal fully covered metallic stent for refractory biliary strictures after living donor liver transplantation: Interim results of a prospective multicenter trial	PICO
Morita et al. 1992 [65]	[Biliary endoprosthesis of malignant biliary obstruction using expandable metallic stent--preliminary clinical evaluation]	PICO
Mukai et al. 2020 [66]	Ways to improve stenting in unresectable malignant distal biliary obstruction: Stent design, intraductal placement, and protective role of an intact papilla?	Study design
Nct. 2020 [67]	Comparison Between Transpapillary and Suprapapillary Metal Stent	Protocol
Nct. 2020 [68]	Intraintestinal Extended Biliary Stents Preventing Duodenobiliary Reflux in Patients With Biliary Stricture	Protocol
Park et al. 2014 [69]	Intraductal placement of modified fully covered metallic stent for distal malignant biliary obstruction	PICO
Park et al. 2019 [70]	Intraductal placement of non-flared fully covered metallic stent for refractory anastomotic biliary strictures after living donor liver transplantation: Long-term results of prospective multicenter trial	PICO
Pécsi et al. 2020 [71]	Are Suprapapillary Biliary Stents Superior to Transpapillary Biliary Stents?	Study design
Peixoto et al. 2017 [72]	Degenerate intraductal papillary mucinous carcinoma with fish-mouth appearance of the papilla	Study design
Sackmann et al. 2016 [73]	Bile duct stenosis: Diagnosis and management by endoscopy	Study design
Sandha et al. 2016 [74]	Clinical utility of biliary metal stent use across a broad range of indications-interim results of a large prospective multi-centre Canadian registry	PICO
Sasahira et al. 2012 [75]	Endoscopic management with inside stent for proximal benign biliary stricture after laparoscopic cholecystectomy	Study design
Sato et al. 2017 [76]	Endoscopic treatment of biliary strictures after adult living donor liver transplantation with duct-to-duct reconstruction	Study design
Sato et al. 2019 [77]	Long-term outcomes of endoscopic treatment for duct-to-duct anastomotic strictures after living donor liver transplantation	Study design
Shin et al. 2019 [78]	Comparison of the clinical outcomes of suprapapillary and transpapillary stent insertion in unresectable cholangiocarcinom a with biliary obstruction	Duplicate
Sung et al. 1992 [79]	Ascending infection of the biliary tract after surgical sphincterotomy and biliary stenting	PICO

Takada et al. 2019 [80]	THE EFFICACY OF SELF-EXPANDABLE METALLIC STENT PLACEMENT ABOVE THE PAPILLA IN THE PATIENTS WITH DISTAL MALIGNANT BILIARY OBSTRUCTION	PICO
Takahashi et al. 2017 [81]	Initial experience of endoscopic stent placement above the Oddi (inside-stent) for preoperative malignant biliary obstruction	PICO
Taniguchi et al 2018 [82]	Intraductal versus transpapillary fully covered self-expandable metal stent placement for malignant biliary strictures	Duplicate
Taniguchi et al 2019 [83]	Intraductal vs. transpapillary fully covered metal stent placement for malignant biliary strictures	Duplicate
Walters et al. 1998 [84]	Current role of endoscopic retrograde cholangiopancreatography in the management of benign pancreatic disease	Study design
Yamauchi et al. 2019 [85]	Intraductal plastic stent placement is an effective therapy for unresec table malignant biliary obstruction	Duplicate
Yamauchi et al. 2016 [86]	Intraductal plastic stent placement is an effective therapy for malignant biliary strictures	Duplicate
Yamauchi et al. 2018 [87]	Temporary non-flared fully covered self-expandable metal stent placement for refractory benign choledochojejunal anastomotic stricture	Study design
Yang et al. 2019 [88]	Hepatobiliary and Pancreatic: Management of a benign bilio-enteric stricture by a novel covered stent with a long lasso	Study design
Yoo et al. 2020 [89]	Intraductal placement of non-flared fully covered metallic stent for refractory anastomotic biliary strictures after living donor liver transplantation: Long-term results of prospective multicenter trial	PICO
Zeissig et al. 2018 [90]	Direct endoscopy and diagnosis of adenocarcinoma following metal stent-based drainage of a pancreatic cyst	Study design

Table S2. Summary of procedure-related complications and endoscopic sphincterotomy rate.

Author, publication date (patient number in supra-/transpapillary group)	Survival (supra- vs. transpapillary)	Sphincterotomy (supra- vs. transpapillary)	Procedure related complications (supra- vs. transpapillary)
**Brijbassie et al. 2015 (112 vs 83) [91]**	N/A	partial almost in all cases vs 83	bleeding: N/A<br>cholecystitis: N/A<br>cholangitis: 7 vs 6<br>pancreatitis: N/A<br>perforation: N/A
**Cho et al. (abstract) 2013 (40 vs 44) [92]**	Mean: 221.8±171.1 vs 284±174.5 days (p=0.414)	0 vs 44	bleeding: 0 vs 0<br>cholecystitis: 1 vs 2<br>cholangitis: 2 vs 2 (p=0.987)<br>pancreatitis: 0 vs 1<br>perforation: N/A
**Cosgrove et al. 2016 (52 vs 120) [93]**	Median: 26 vs 29 weeks (p= 0.49)	5 vs 108	bleeding: 0 vs 2<br>cholecystitis: N/A<br>cholangitis: 0 vs 1<br>pancreatitis: 0 vs 9 (p=0.059)<br>perforation: 1 vs 2
**Inatomi et al. 2013 (12 vs. 17) [94]**	N/A	In metal stents	bleeding: 0 vs 0<br>cholecystitis: N/A<br>cholangitis: N/A<br>pancreatitis: N/A<br>perforation: 0 vs 0
**Kobayashi et al. 2015 (25 vs 32) [95]**	N/A	3 vs 3	bleeding: 1 vs. 0<br>cholecystitis: N/A<br>cholangitis: 0 vs. 0<br>pancreatitis: 1 vs. 4<br>perforation: N/A
**Kubota et al. 2016 (17 vs 23) [96]**	24.8 vs. 20.4 months (p= 0.327)	0 vs. 4	bleeding: N/A<br>cholecystitis: N/A<br>cholangitis: 7 vs 20<br>pancreatitis: 2 vs 1<br>perforation: N/A
**Lee et al. 2018 (abstract) (27 vs 29) [97]**	N/A	N/A	N/A
**Pedersen et al. 1998 (17 vs 17) [98]**	Median (IQR): 144 (82-347) vs<br>46 (35-155) days	0 vs. 0	bleeding: 0 vs 0<br>cholecystitis: 0 vs 0<br>cholangitis: 1 vs 3<br>pancreatitis: 0 vs 0<br>perforation: 0 vs 0
**Shin et al. 2020 (44 vs 29) [99]**	Cumulative patient survival at 1, 6, 12 months: 91% vs 90%, 43% vs 35%, 11% vs 17%	44 vs 29	bleeding: N/A<br>cholecystitis: 2 vs 0<br>cholangitis: 6 vs 5<br>pancreatitis: 0 vs 2<br>perforation: N/A
**Takada et al. 2020 (30 vs 43) [100]**	Median: 269 days vs 302 days (p= 0.891)	10 vs 12	bleeding: N/A<br>cholecystitis: 0 vs 2<br>cholangitis: N/A

			pancreatitis: N/A<br>perforation: N/A
**Taniguchi et al. 2020 (abstract) (38 vs 58) [101]**	N/A	N/A	N/A
**Uchida et al. 2005 (16 vs 16) [102]**	N/A	2 vs. 1	bleeding: 0 vs. 0<br>cholecystitis: 0 vs 0<br>cholangitis: 0 vs 0<br>pancreatitis: 0 vs 0<br>perforation: 0 vs. 0
**Yamaguchi et al. 2019 (abstract) (40 vs 46) [103]**	N/A	N/A	bleeding: 0 vs. 0<br>cholecystitis: 3 vs N/A<br>cholangitis: 0 vs 0<br>pancreatitis: 0 vs 0<br>perforation: N/A

N/A: not available.

Table S3. Summary of findings table comprising the level of evidence for stent patency, stent migration, post-ERCP cholangitis, and pancreatitis.

Outcomes	Anticipated absolute effects* (95% CI)		Relative effect (95% CI)	№ of participants (studies)	Certainty of the evidence (GRADE)	Comments
	Risk with transpapillary stent	Risk with suprapapillary stent				
Patency assessed with: WMD		WMD 47.2 more (18.75 more to 75.64 more)	-	971 (11 observational studies) [91-96, 98-100, 102]	⊕⊕◯◯ Low	Suprapapillary biliary stent was associated with longer stent patency.
Stent migration assessed with: OR	10 per 100	7 per 100 (2 to 25)	OR 0.66 (0.15 to 2.94)	376 (7 observational studies) [92, 95-98, 100, 102]	⊕◯◯◯ Very low	No significant difference appeared concerning the stent migration between the suprapapillary and transpapillary stent positions.
Post-ERCP cholangitis assessed with: OR	12 per 100	6 per 100 (3 to 13)	OR 0.52 (0.25 to 1.10)	598 (6 observational studies) [91-93, 96, 98, 99]	⊕◯◯◯ Very low	No significant difference appeared concerning the post-ERCP cholangitis rate between the suprapapillary and transpapillary stent positions.
Post-ERCP pancreatitis assessed with: OR	7 per 100	3 per 100 (1 to 9)	OR 0.38 (0.11 to 1.28)	426 (5 observational studies) [92, 93, 95, 96, 99]	⊕◯◯◯ Very low	No significant difference appeared concerning the post-ERCP pancreatitis rate between the suprapapillary and transpapillary stent positions.

*The risk in the intervention group (and its 95% confidence interval) is based on the assumed risk in the comparison group and the relative effect of the intervention (and its 95% CI). WMD: weighted mean difference; CI: confidence interval; OR: odds ratio.

Table S4. Summary of findings table comprising the level of evidence for the subgroup of metal stents.

Outcomes	Anticipated absolute effects* (95% CI)		Relative effect (95% CI)	№ of participants (studies)	Certainty of the evidence (GRADE)	Comments
	Risk with transpapillary stent	Risk with suprapapillary stent				
Patency assessed with: WMD		WMD 10.18 days more (38.83 fewer to 59.18 more)	-	597 (5 observational studies) [91-93, 99, 100]	⨁◯◯◯ Very low	No significant difference appeared concerning the stent patency between the suprapapillary and transpapillary stent positions.
Post-ERCP cholangitis assessed with: OR	5 per 100	4 per 100 (2 to 9)	OR 0.85 (0.40 to 1.81)	524 (4 observational studies) [91-93, 99]	⨁◯◯◯ Very low	No significant difference appeared concerning the rate of post-ERCP cholangitis between the suprapapillary and transpapillary stent positions.
Post-ERCP pancreatitis assessed with: OR	6 per 100	1 per 100 (0 to 6)	OR 0.16 (0.03 to 0.95)	329 (3 observational studies) [92, 93, 99]	⨁◯◯◯ Very low	Significantly lower rate of post-ERCP pancreatitis appeared in the suprapapillary stent position group.

*The risk in the intervention group (and its 95% confidence interval) is based on the assumed risk in the comparison group and the relative effect of the intervention (and its 95% CI). WMD: weighted mean difference; CI: confidence interval; OR: odds ratio.

Table S5. Summary of findings table comprising the level of evidence for the subgroup of plastic stents.

Outcomes	**Anticipated absolute effects*** (95% CI)		Relative effect (95% CI)	№ of participants (studies)	Certainty of the evidence (GRADE)	Comments
	**Risk with transpapillary stent**	**Risk with suprapapillary stent**				
Patency assessed with: WMD		WMD **78.45 days more** (41.42 more to 115.47 more)	-	278 (6 observational studies) [94-96, 98, 102, 103]	⊕◯◯◯ Very low	Suprapapillary biliary stent was associated with longer stent patency.
Stent migration assessed with: OR	11 per 100	**17 per 100** (3 to 60)	OR 1.57 (0.22 to 11.47)	163 (4 observational studies) [95, 96, 98, 102]	⊕◯◯◯ Very low	No significant difference appeared concerning the stent migration between the suprapapillary and transpapillary stent positions.

***The risk in the intervention group** (and its 95% confidence interval) is based on the assumed risk in the comparison group and the **relative effect** of the intervention (and its 95% CI). WMD: weighted mean difference; **CI:** confidence interval; **OR:** odds ratio.

Table S6. Summary of findings table comprising the level of evidence for the subgroup of full text included.

Outcomes	Anticipated absolute effects* (95% CI)		Relative effect (95% CI)	№ of participants (studies)	Certainty of the evidence (GRADE)	Comments
	Risk with transpapillary stent	Risk with suprapapillary stent				
Patency assessed with: WMD		WMD 60.74 days more (14.1 more to 107.38 more)	-	510 (8 observational studies) [93-96, 98-100, 102]	⊕⊕◯◯ Low	Suprapapillary biliary stent was associated with longer stent patency.
Post-ERCP cholangitis assessed with: OR	15 per 100	6 per 100 (2 to 15)	OR 0.34 (0.12 to 0.99)	319 (4 observational studies) [93, 96, 98, 99]	⊕◯◯◯ Very low	Significantly lower rate of post-ERCP cholangitis appeared in the suprapapillary stent position group.
Post-ERCP pancreatitis assessed with: OR	8 per 100	3 per 100 (1 to 13)	OR 0.37 (0.08 to 1.69)	342 (4 observational studies) [93, 95, 96, 99]	⊕◯◯◯ Very low	No significant difference appeared concerning the post-ERCP pancreatitis rate between the suprapapillary and transpapillary stent positions.

*The risk in the intervention group (and its 95% confidence interval) is based on the assumed risk in the comparison group and the relative effect of the intervention (and its 95% CI). WMD: weighted mean difference; CI: confidence interval; OR: odds ratio.

Figures

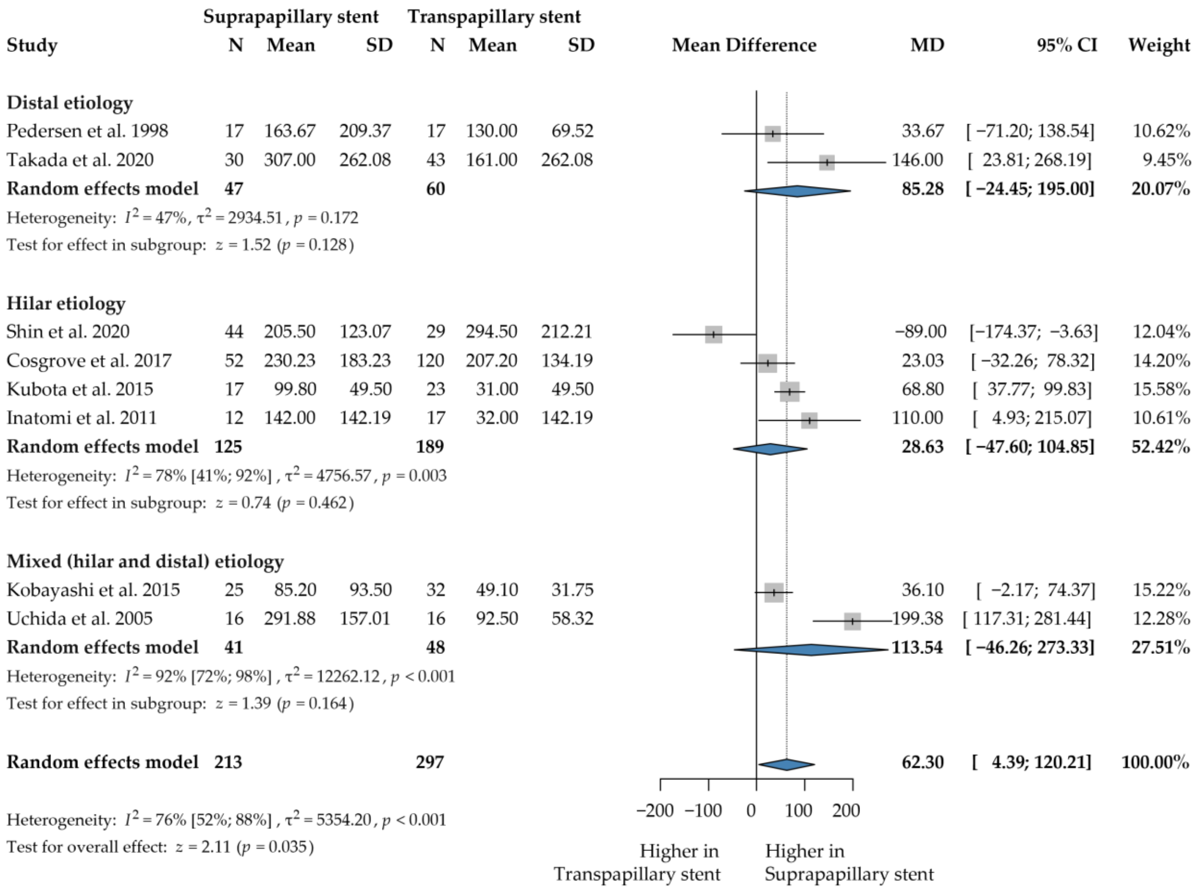

Figure S1. Forest plot comparing stent patency time between suprapapillary and transpapillary stents, including full texts with malignant indications. WMD: weighted mean difference; p: P-value; CI: confidence interval; I-squared: I^2. Unit of measurement: day. [93-96, 98-100, 102]

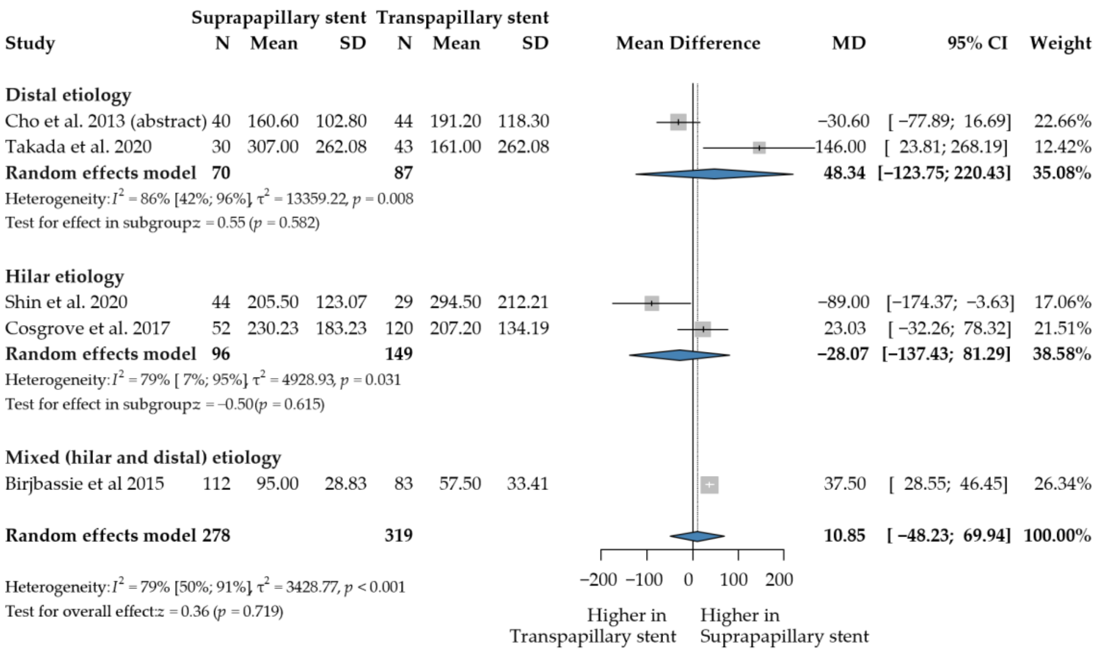

Figure S2. Forest plot comparing stent patency time between suprapapillary and transpapillary metal stents. WMD: weighted mean difference; p: P-value; CI: confidence interval; I-squared: I^2. Unit of measurements: day. [91-93, 99, 100]

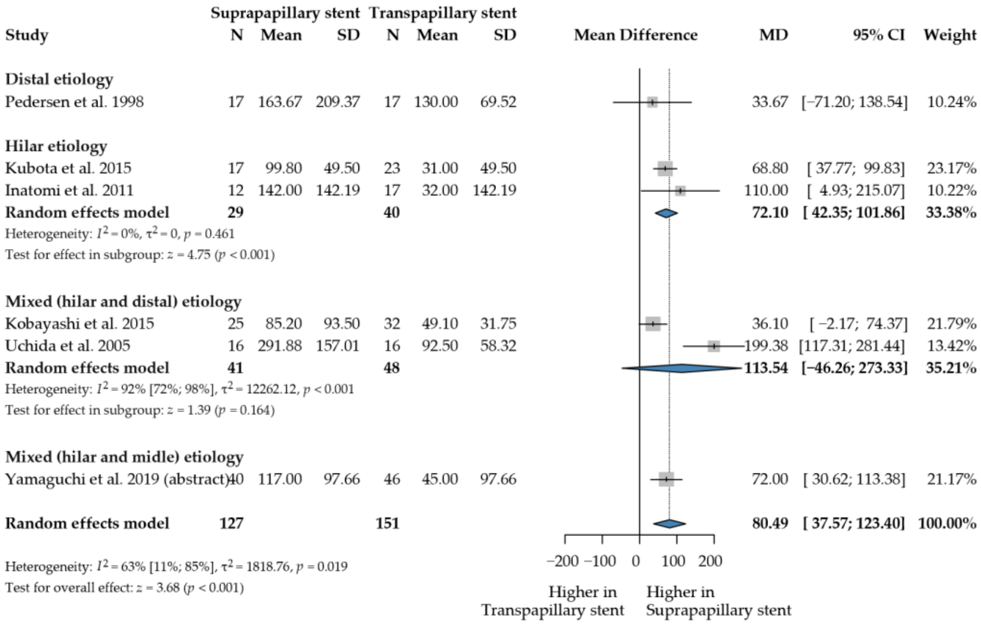

Figure S3. Forest plot comparing stent patency time between suprapapillary and transpapillary plastic stents. WMD: weighted mean difference; p: P-value; CI: confidence interval; I-squared: I^2. Unit of measurement: day. [94-96, 98, 102, 103]

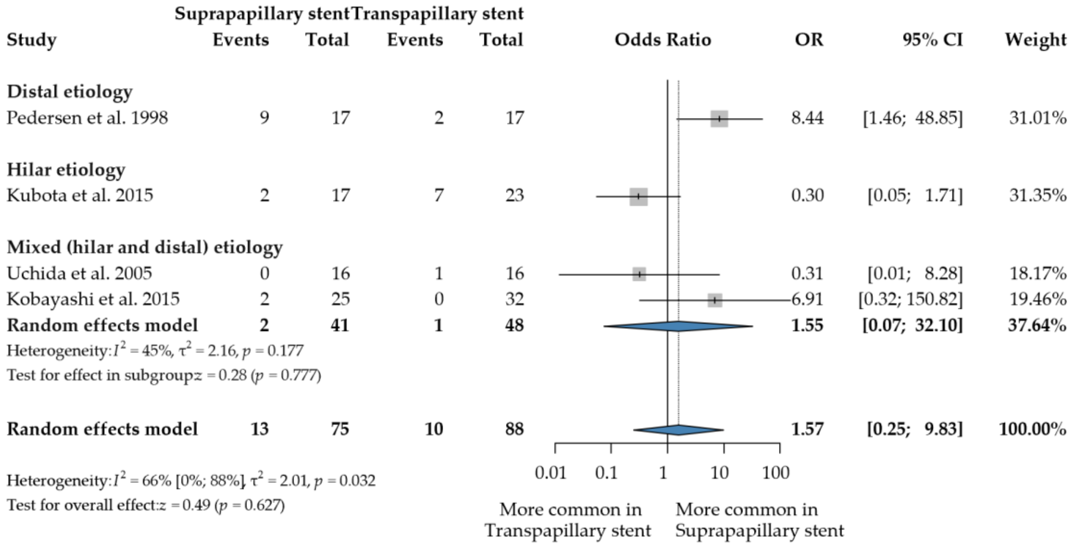

Figure S4. Forest plot comparing stent migration rate between suprapapillary and transpapillary plastic stents. OR: odds ratio, p: P-value; CI: confidence interval; I-squared: I^2. [95, 96, 98, 102]

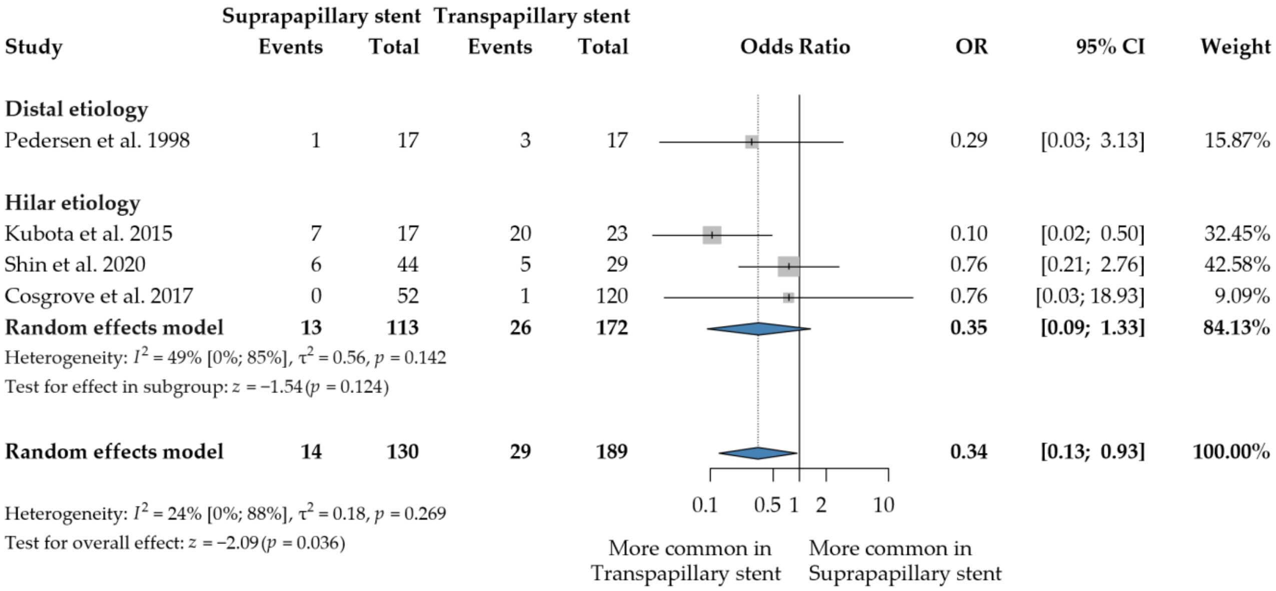

Figure S5. Forest plot comparing cholangitis rate between suprapapillary and transpapillary stents, including full texts with malignant indications. OR: odds ratio, p: P-value; CI: confidence interval; I-squared: I^2. [93, 96, 98, 99]

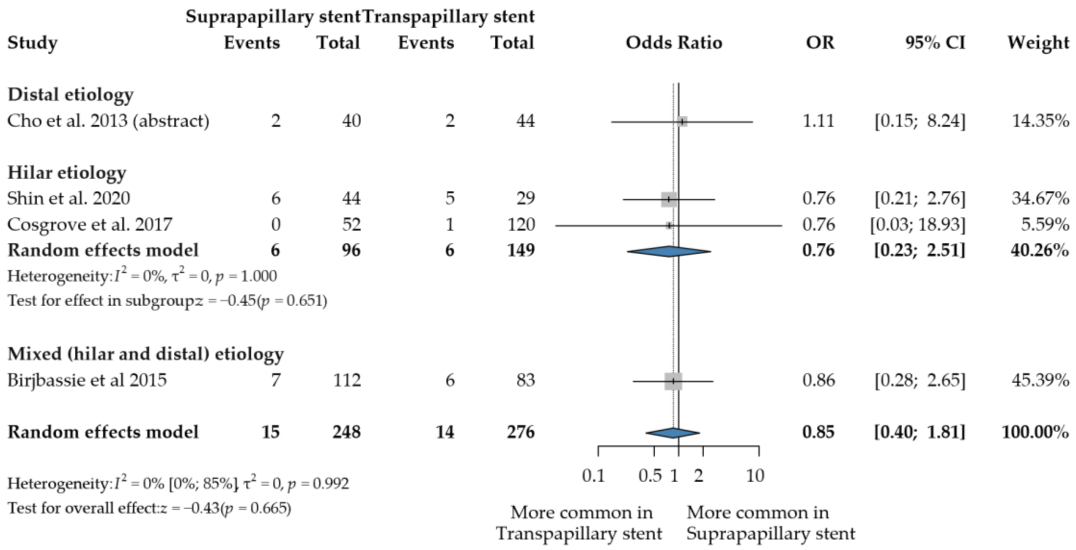

Figure S6. Forest plot comparing cholangitis rate between suprapapillary and transpapillary metal stents. OR: odds ratio, p: P-value; CI: confidence interval; I-squared: I^2. [91-93, 99]

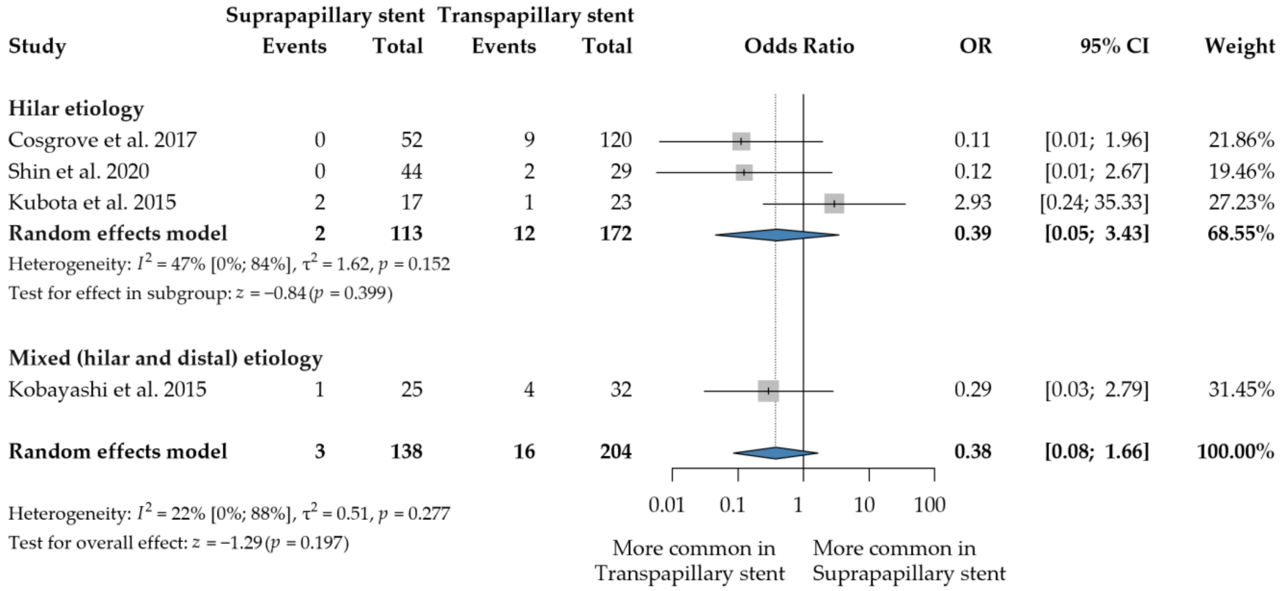

Figure S7. Forest plot comparing pancreatitis rate between suprapapillary and transpapillary stents including full texts. OR: odds ratio, p: P-value; CI: confidence interval; I-squared: I². [93, 95, 96, 99]

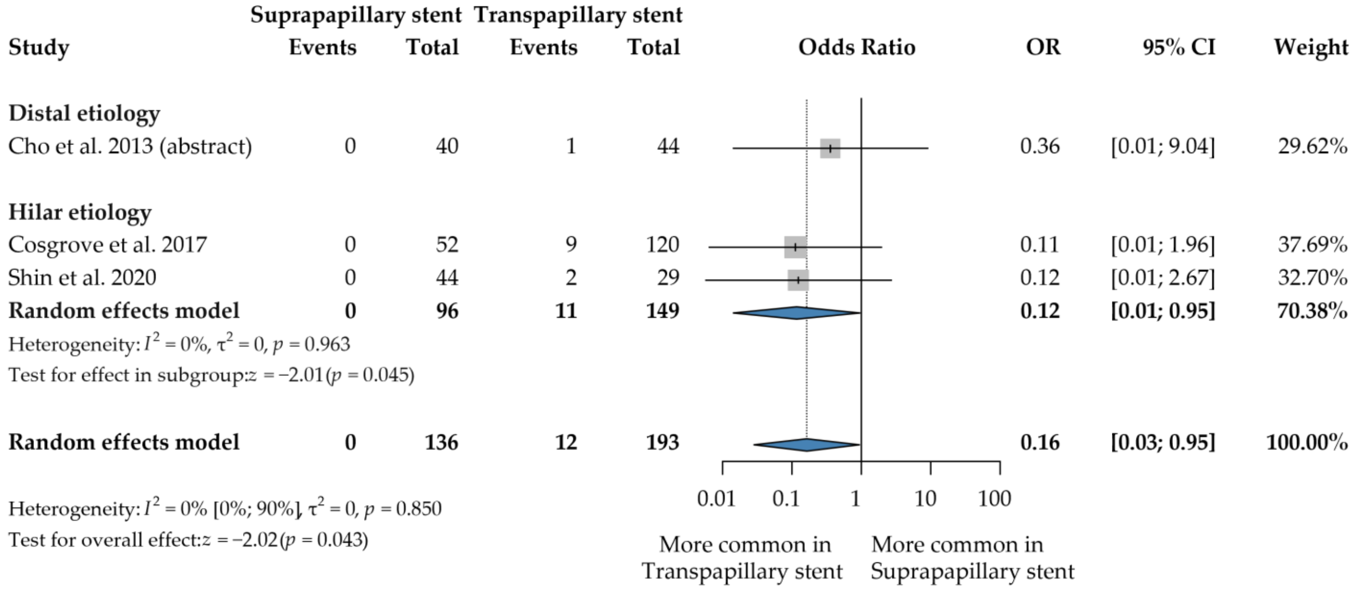

Figure S8. Forest plot comparing pancreatitis rate between suprapapillary and transpapillary metal stents. OR: odds ratio, p: P-value; CI: confidence interval; I-squared: I². [92, 93, 99]

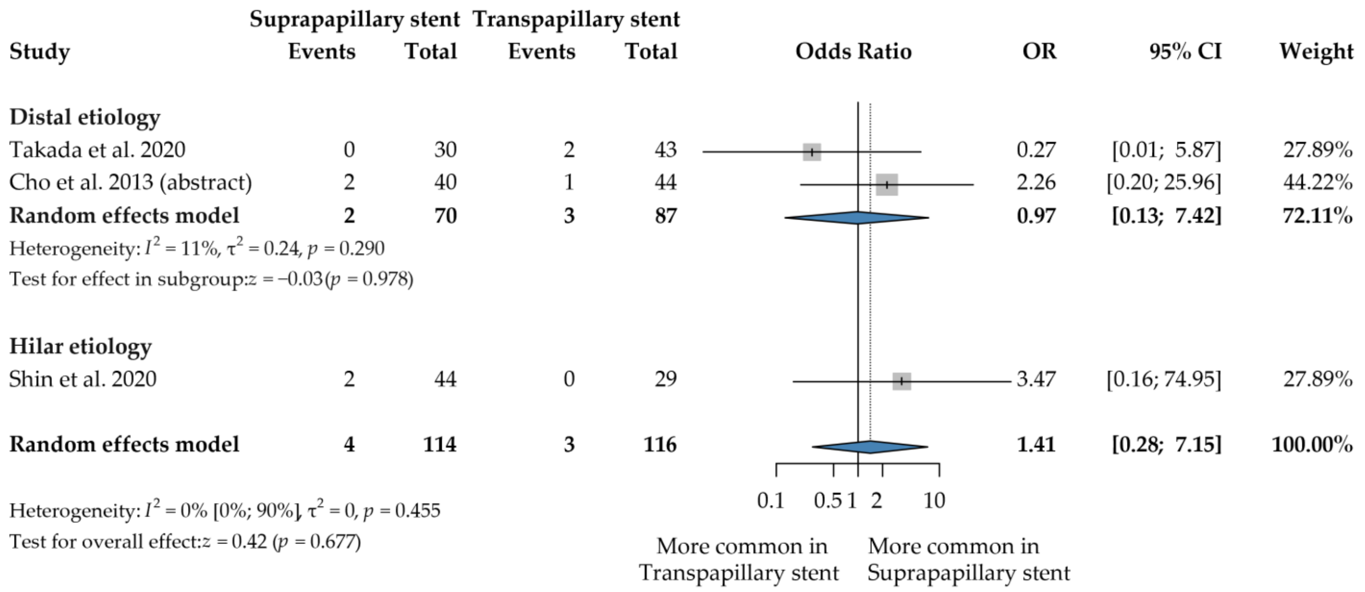

Figure S9. Forest plot comparing cholecystitis rate between suprapapillary and transpapillary metal stents. OR: odds ratio, p: P value; CI: confidence interval; I-squared: I². [92, 99, 100]

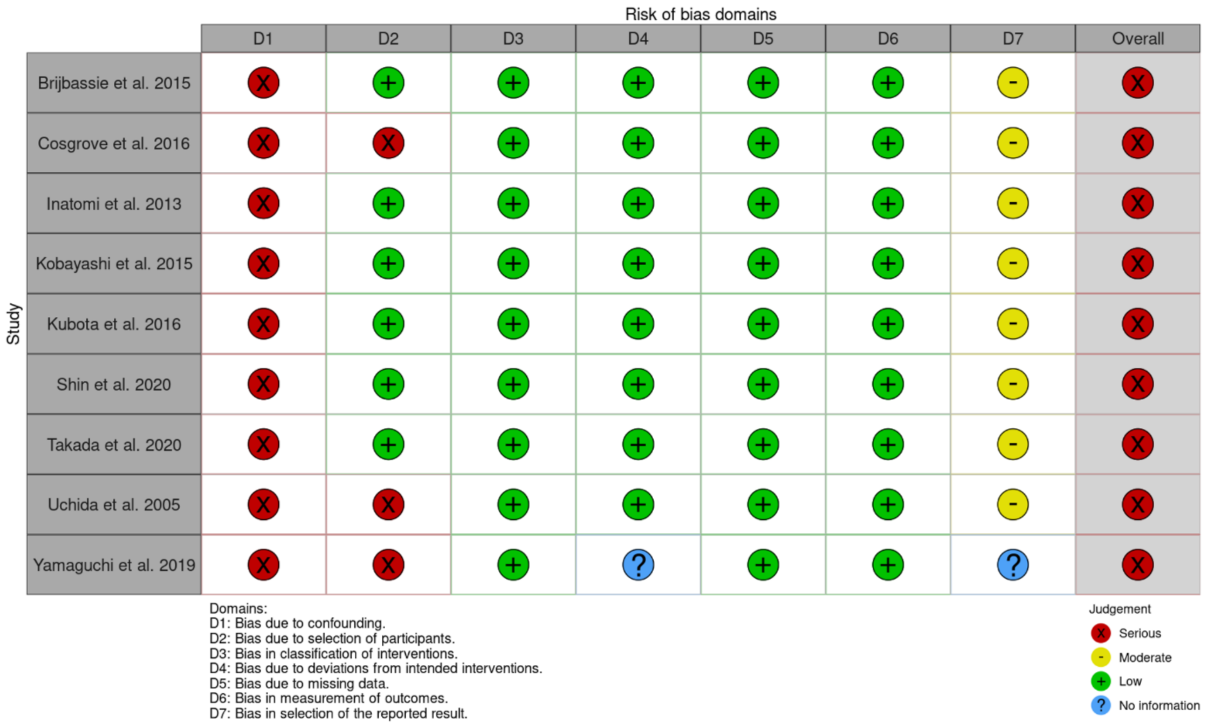

Figure S10. Risk of Bias Assessment (study-level judgments) in observational studies with the Risk Of Bias In Non-Randomized Studies - of Interventions (ROBINS-I). Outcome: Stent patency time. [91, 93-96, 99, 100, 102, 103]

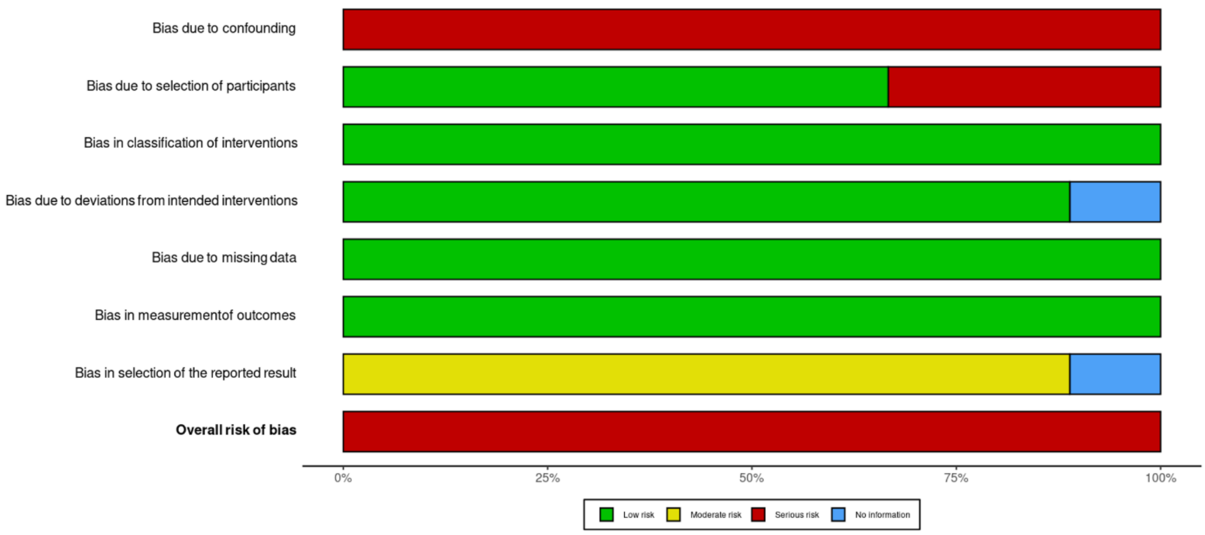

Figure S11. Risk of bias assessment (domain-level judgments) in observational studies with the Risk Of Bias In Non-Randomized Studies - of Interventions (ROBINS-I). Outcome: Stent patency time.[91, 93-96, 99, 100, 102, 103]

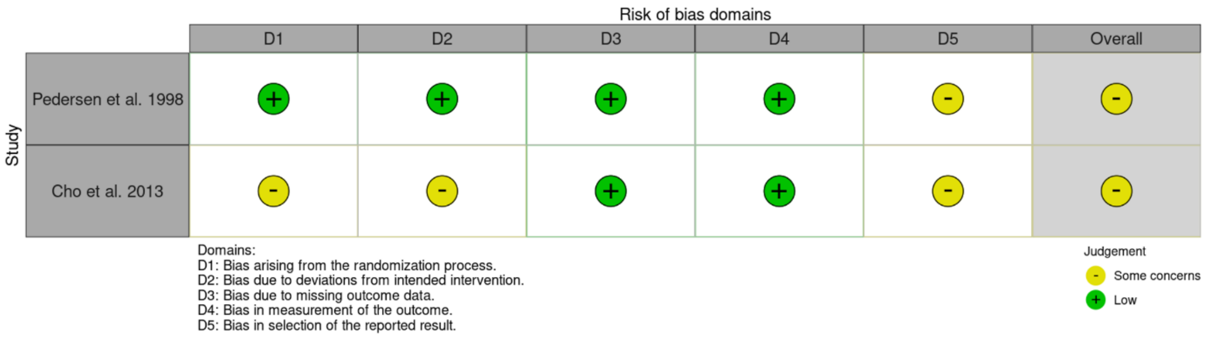

Figure S12. Risk of Bias Assessment (study-level judgments) with version 2 of the Cochrane risk-of-bias tool for randomized trials (RoB 2). Outcome: Stent patency time. [92, 98]

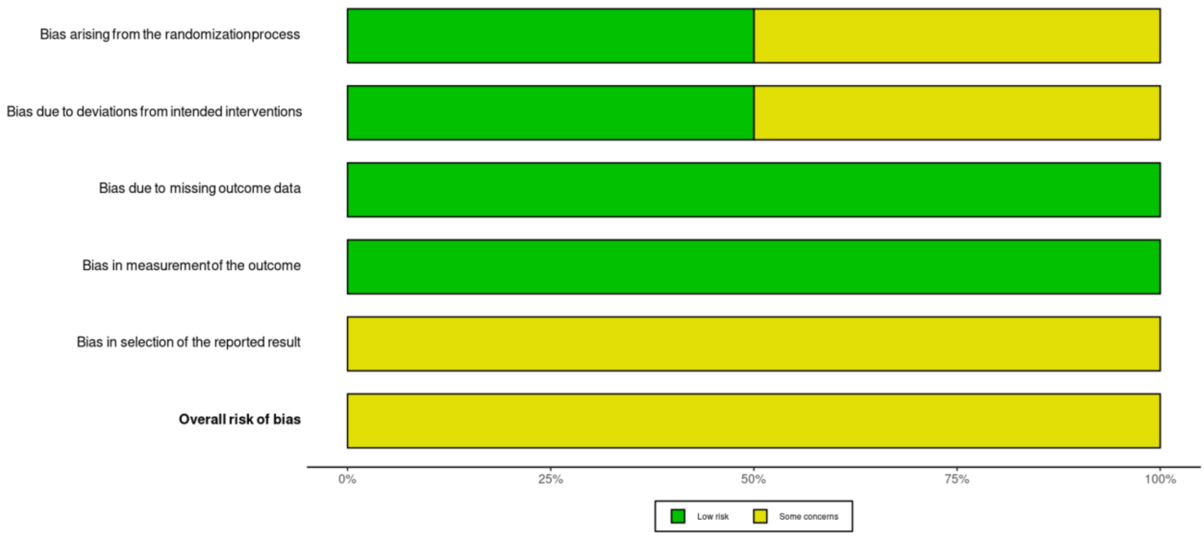

Figure S13. Risk of bias assessment (domain-level judgments) with version 2 of the Cochrane Risk-of-Bias tool for randomized trials (RoB 2). Outcome: Stent patency time. [92, 98]

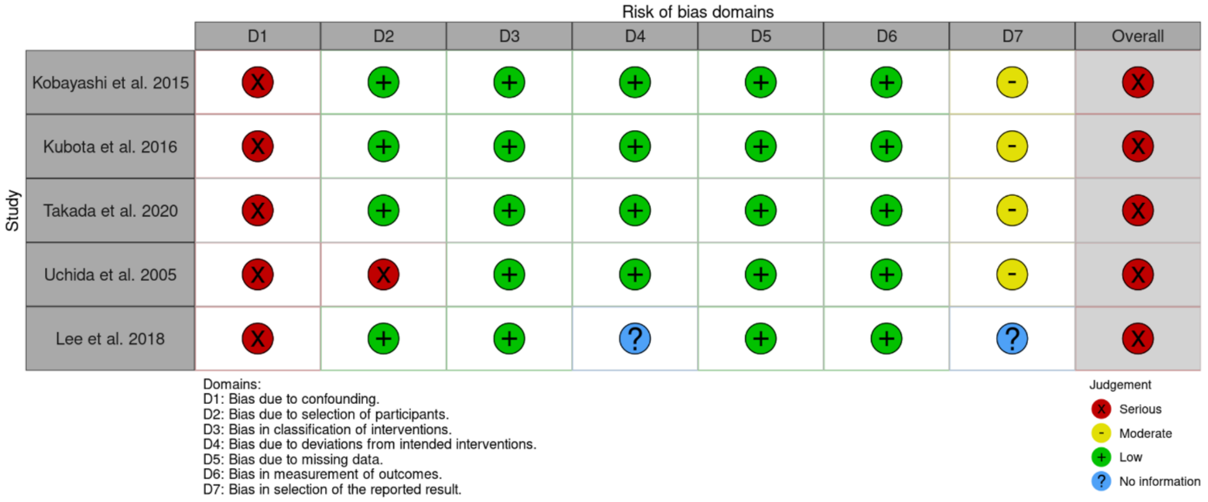

Figure S14. Risk of Bias Assessment (study-level judgments) in observational studies with the Risk Of Bias In Non-Randomized Studies - of Interventions (ROBINS-I). Outcome: Stent stent migration. [95-97, 100, 102]

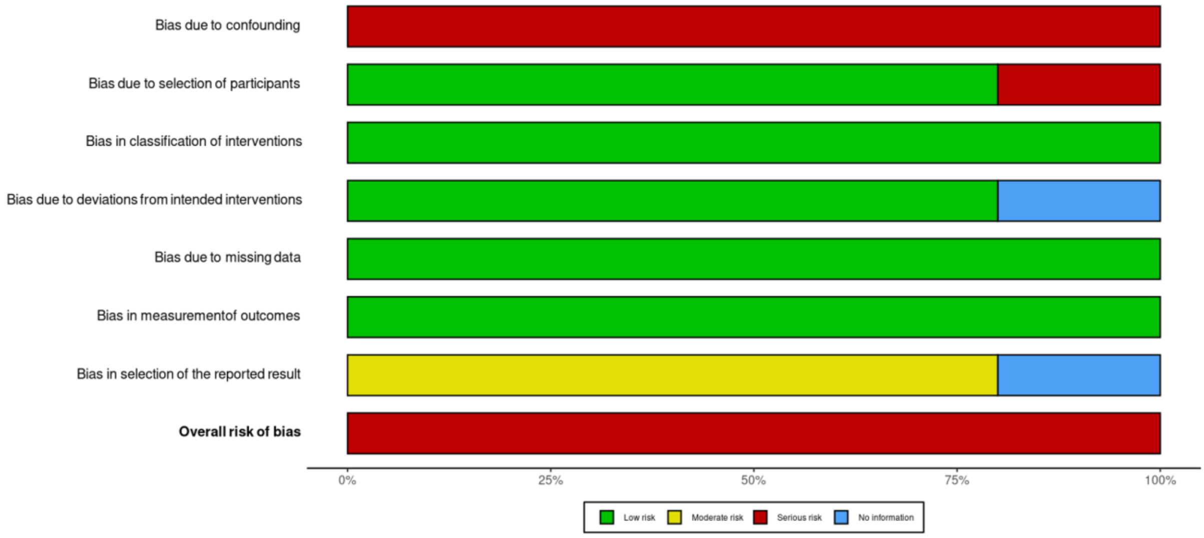

Figure S15. Risk of bias assessment (domain-level judgments) in observational studies with the Risk Of Bias In Non-Randomized Studies - of Interventions (ROBINS-I). Outcome: Stent stent migration. [95-97, 100, 102]

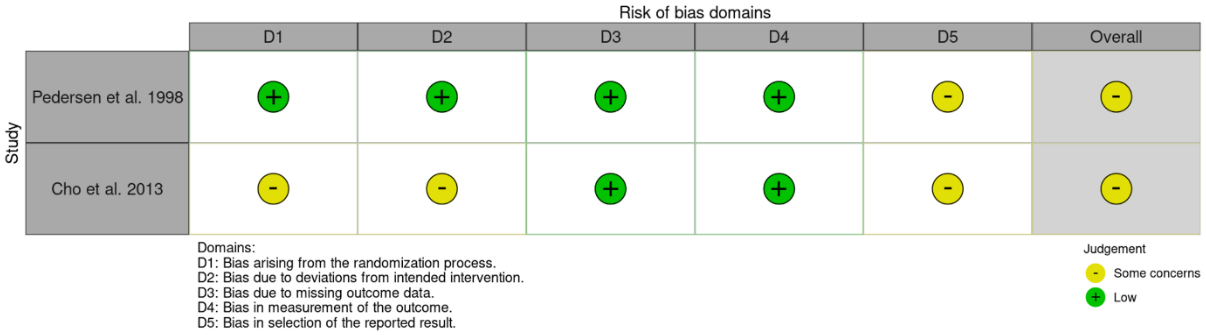

Figure S16. Risk of Bias Assessment (study-level judgments) with version 2 of the Cochrane Risk-of-Bias tool for randomized trials (RoB 2). Outcome: Stent stent migration. [92, 98]

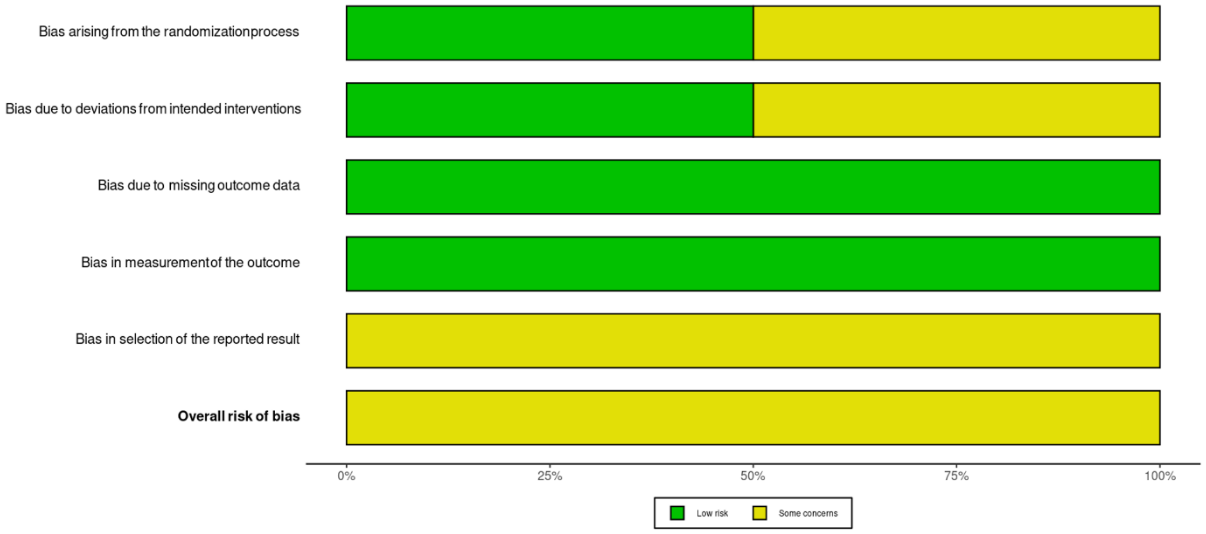

Figure S17. Risk of bias assessment (domain-level judgments) with version 2 of the Cochrane Risk-of-Bias tool for randomized trials (RoB 2). Outcome: Stent stent migration. [92, 98]

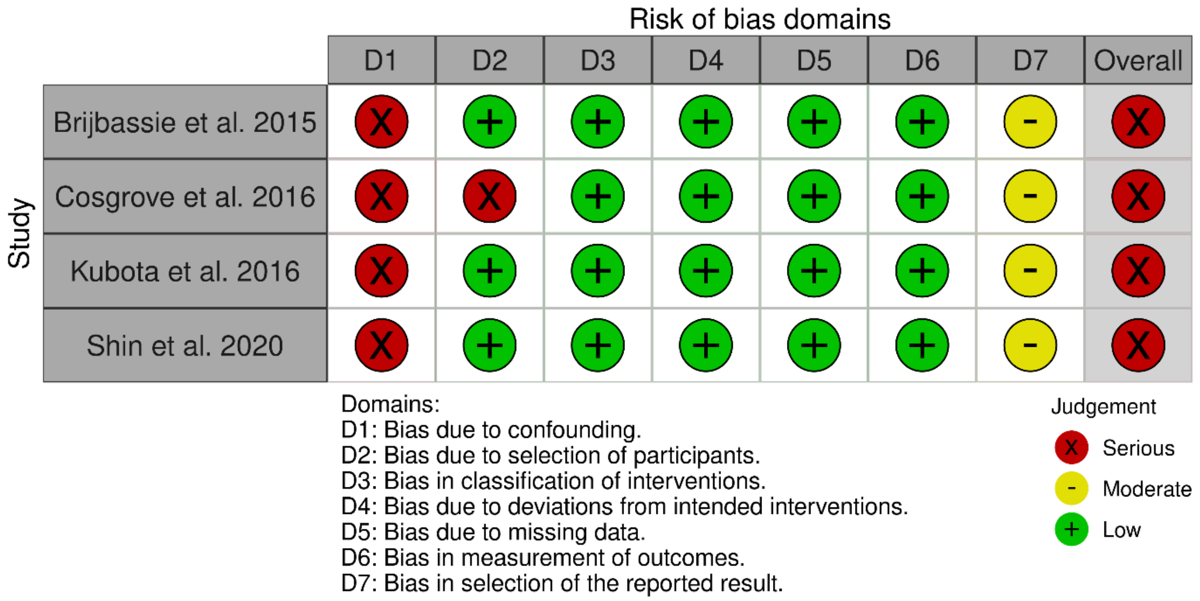

Figure S18. Risk of Bias Assessment (study-level judgments) in observational studies with the Risk Of Bias In Non-Randomized Studies - of Interventions (ROBINS-I). Outcome: cholangitis. [91, 93, 96, 99]

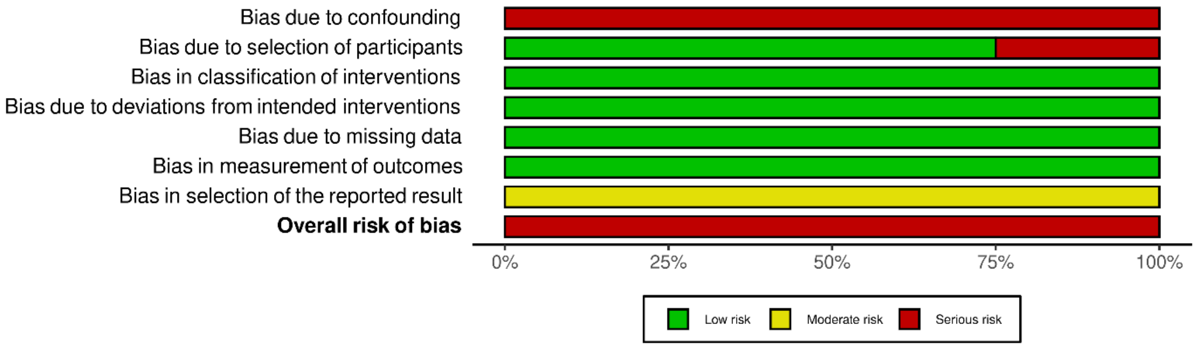

Figure S19. Risk of bias assessment (domain-level judgments) in observational studies with the Risk Of Bias In Non-Randomized Studies - of Interventions (ROBINS-I). Outcome: cholangitis. [91, 93, 96, 99]

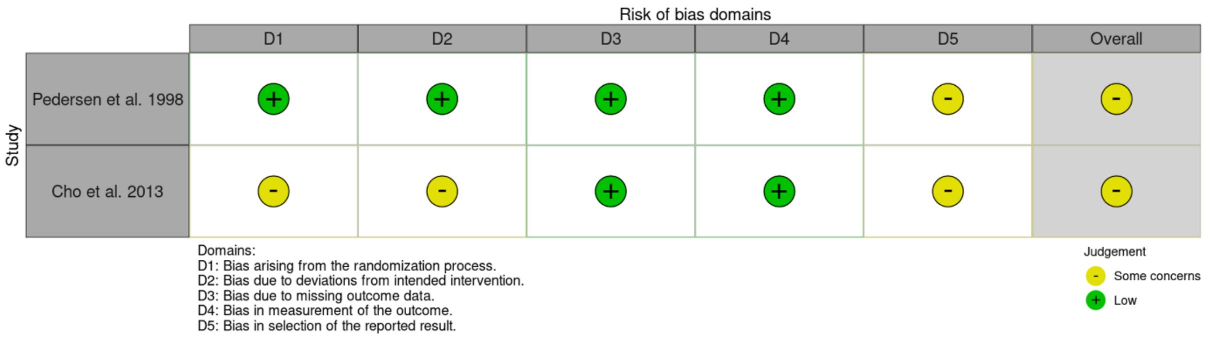

Figure S20. Risk of Bias Assessment (study-level judgments) with version 2 of the Cochrane Risk-of-Bias tool for randomized trials (RoB 2). Outcome: cholangitis. [92, 98]

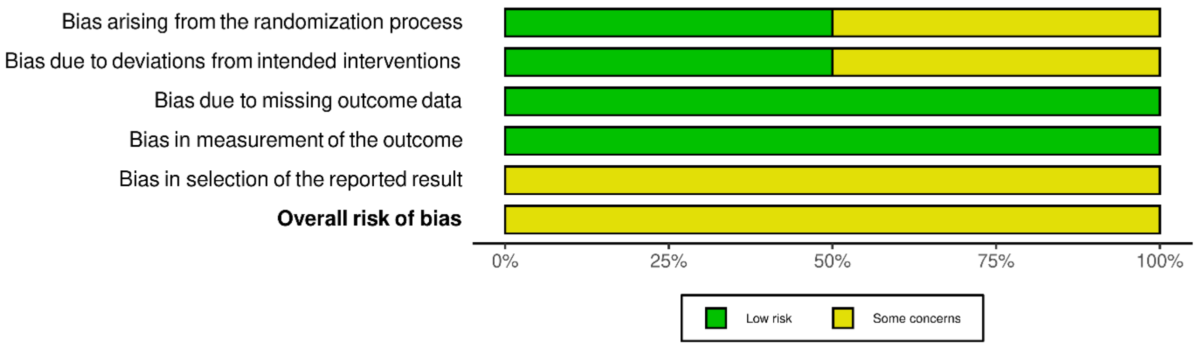

Figure S21. Risk of bias assessment (domain-level judgments) with version 2 of the Cochrane risk-of-bias tool for randomized trials (RoB 2). Outcome: cholangitis. [92, 98]

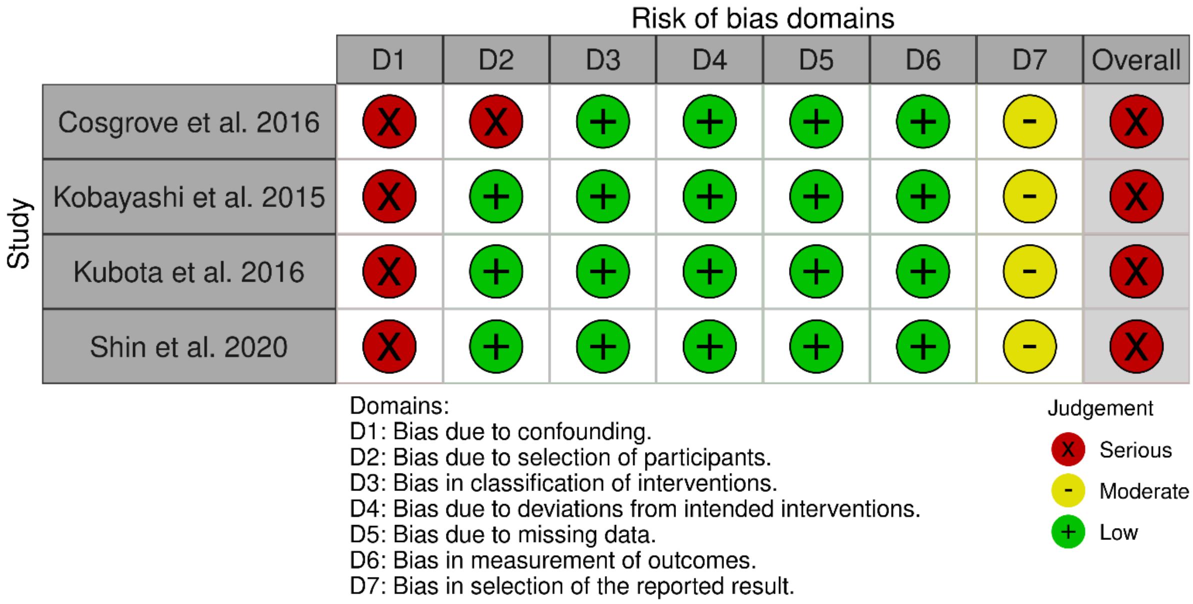

Figure S22. Risk of Bias Assessment (study-level judgments) in observational studies with the Risk Of Bias In Non-Randomized Studies - of Interventions (ROBINS-I). Outcome: pancreatitis. [93, 95, 96, 99]

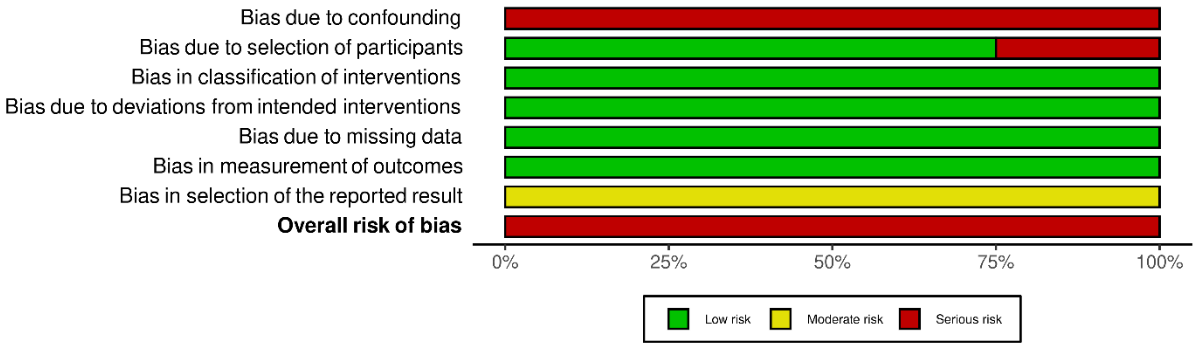

Figure S23. Risk of bias assessment (domain-level judgments) in observational studies with the Risk Of Bias In Non-Randomized Studies - of Interventions (ROBINS-I). Outcome: pancreatitis. [93, 95, 96, 99]

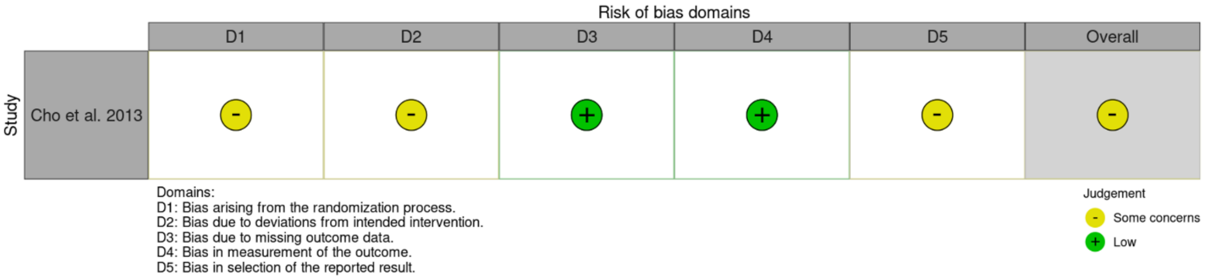

Figure S24. Risk of Bias Assessment (study-level judgments) with version 2 of the Cochrane Risk-of-Bias tool for randomized trials (RoB 2). Outcome: pancreatitis. [92]

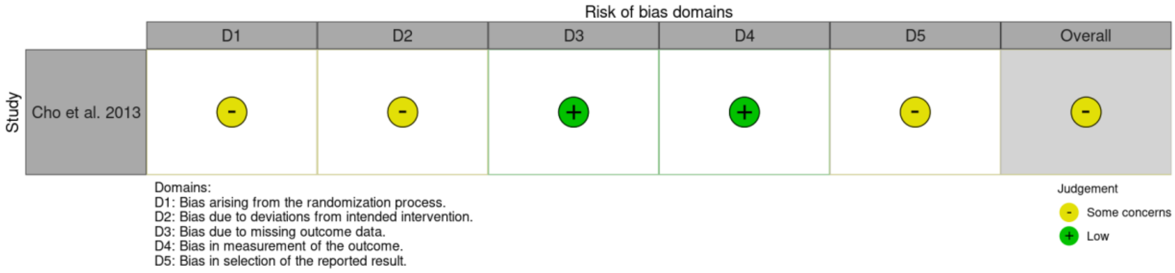

Figure S25. Risk of Bias Assessment (study-level judgements) with the version 2 of the Cochrane risk-of-bias tool for randomized trials (RoB 2). Outcome: Cholecystitis. [92]

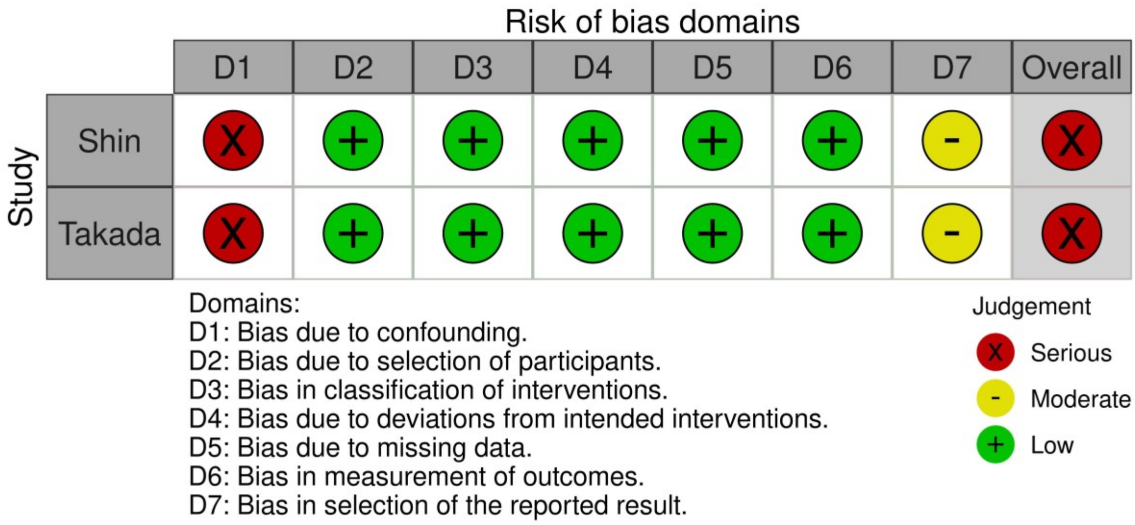

Figure S26. Risk of Bias Assessment (study-level judgements) with the Risk of Bias In Non-Randomized Studies - of Interventions (ROBINS-I). Outcome: Cholecystitis. [99, 100]

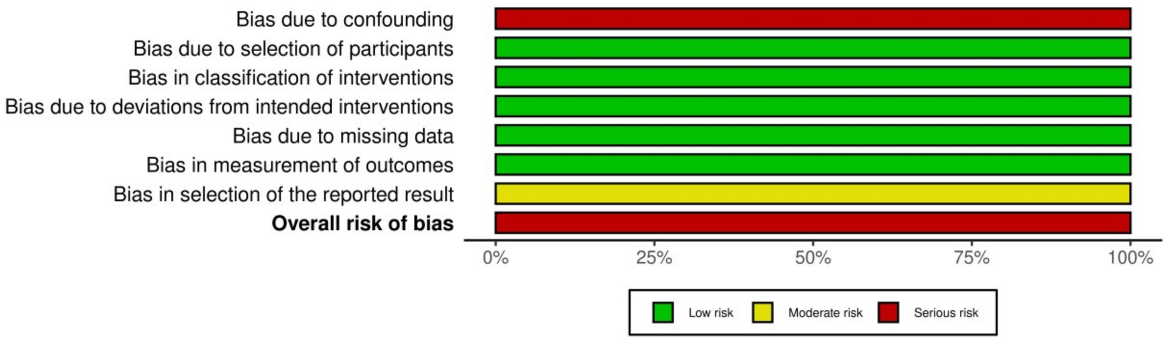

Figure S27. Risk of Bias Assessment (domain-level judgements) with the Risk of Bias In Non-Randomized Studies - of Interventions (ROBINS-I). Outcome: Cholecystitis. [99, 100]

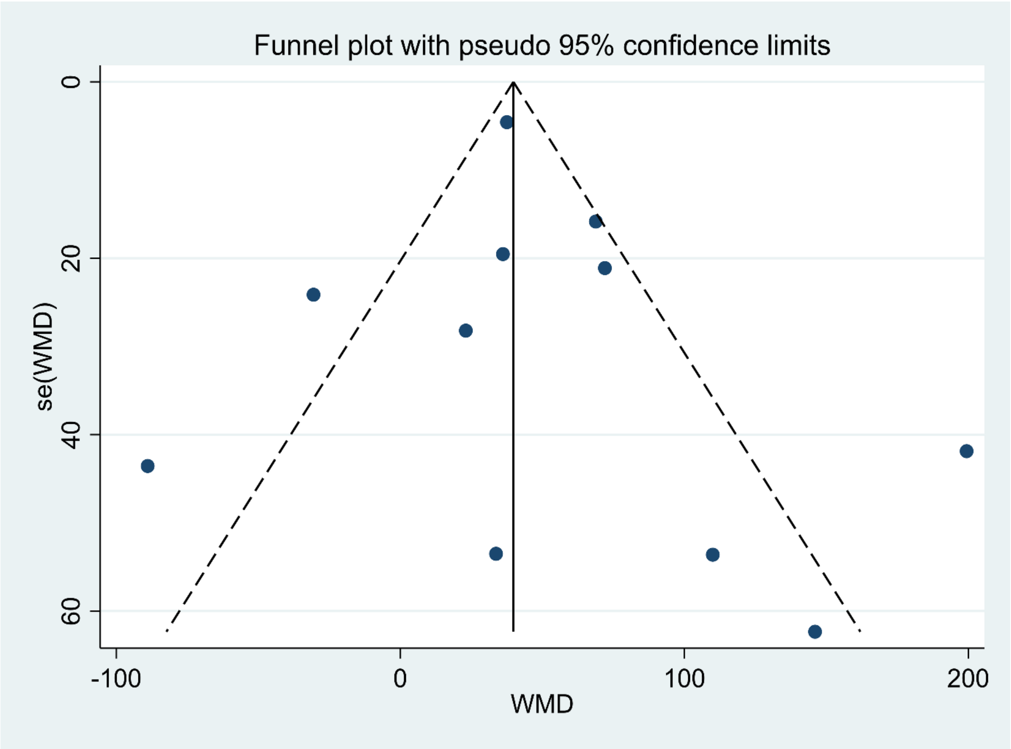

Figure S28. Funnel plot investigating publication bias for the outcome of stent patency (all included publications). Egger's test: p=0.591. [91-96, 98-100, 102, 103]

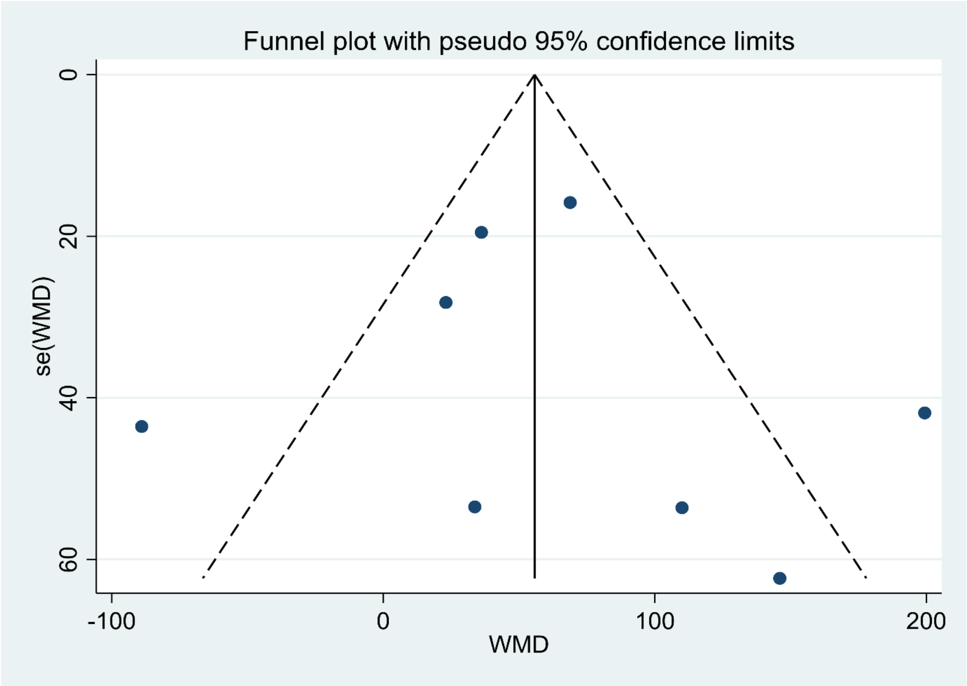

Figure S29. Funnel plot investigating publication bias for the outcome of stent patency (full-texts investigating malignant indication). [93-96, 98-100, 102]

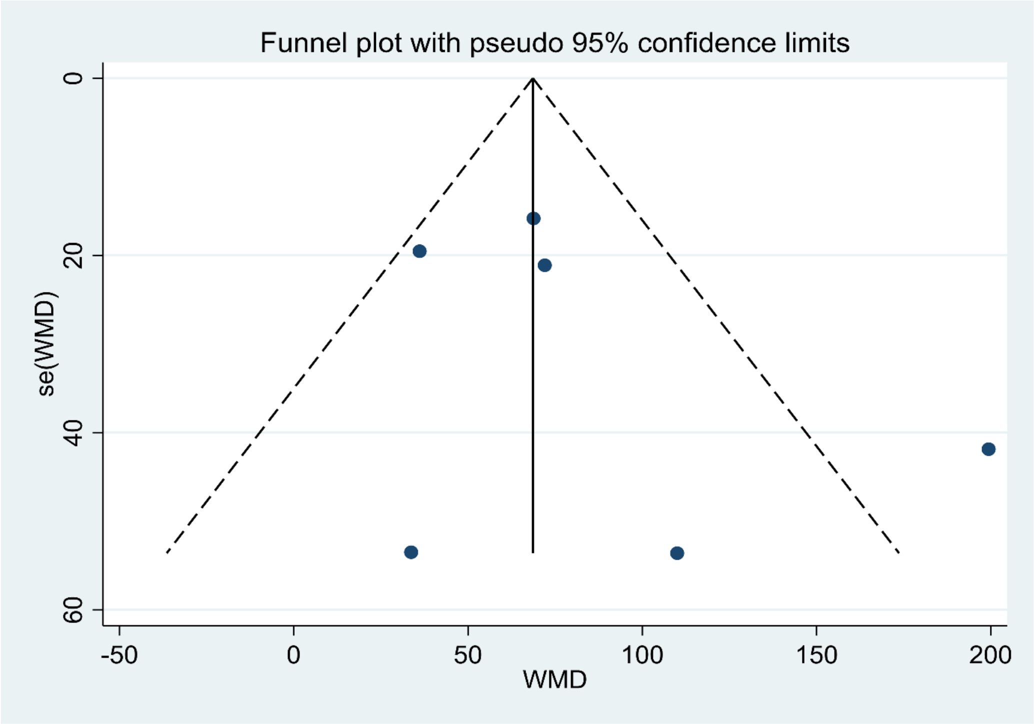

Figure S30. Funnel plot investigating publication bias for the outcome of stent patency (plastic stent). [94-96, 98, 102, 103]

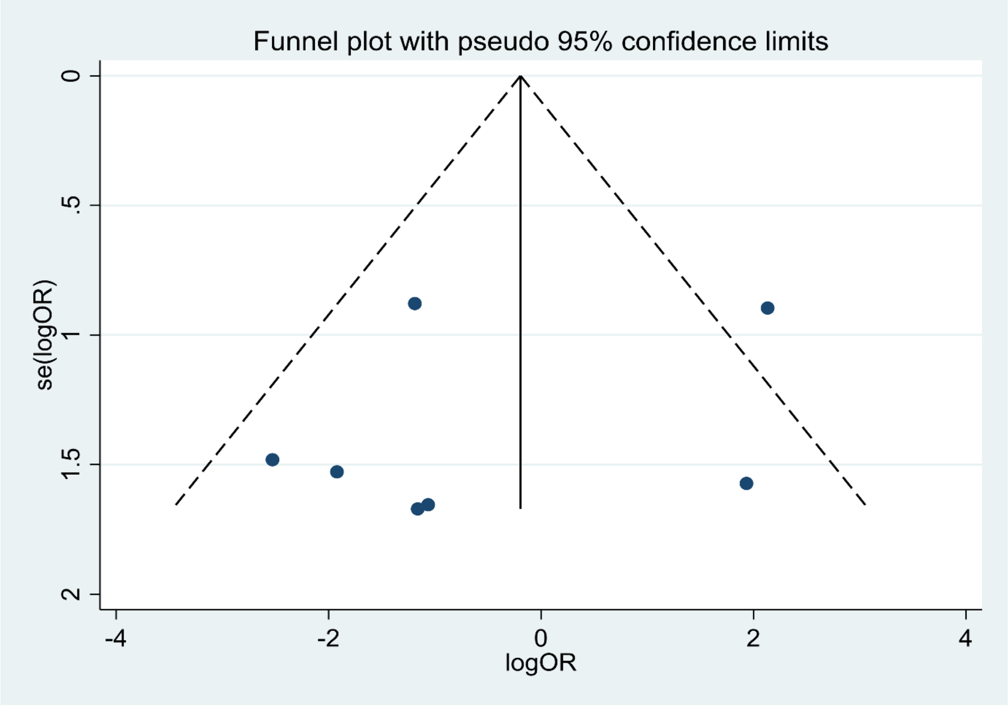

Figure S31. Funnel plot investigating publication bias for the outcome of stent migration rate (all publications). [92, 95-98, 100, 102]

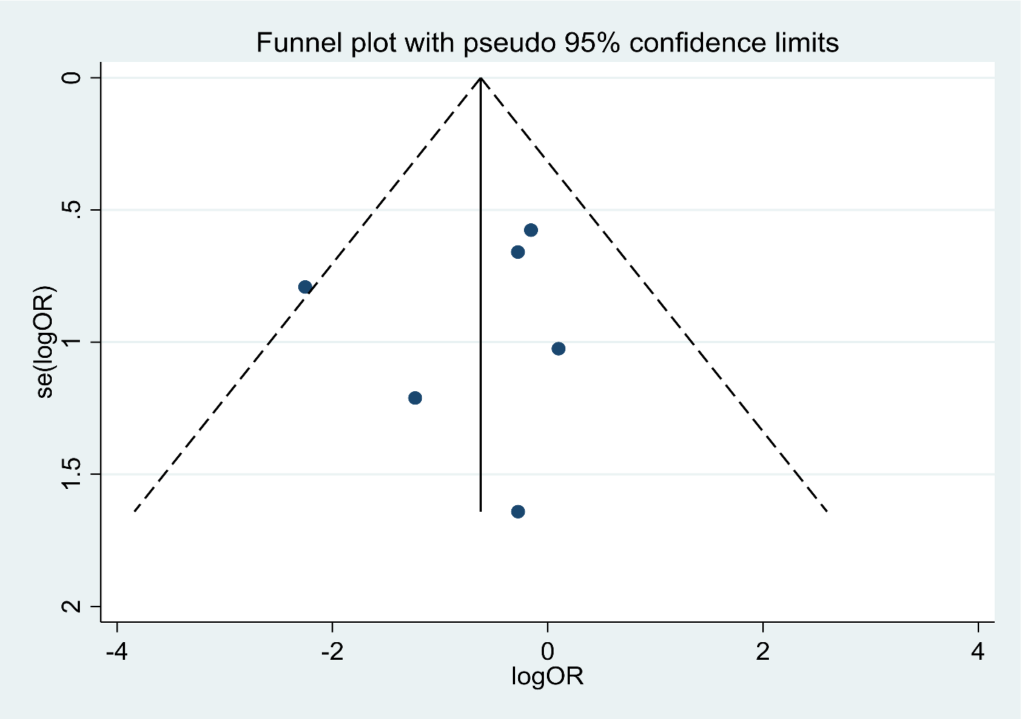

Figure S32. Funnel plot investigating publication bias for the outcome of post-ERCP cholangitis rate (all publications). [91-93, 96, 98, 99]

References:

1. 1. Aepli P, St John A, Gupta S et al. Success and complications of an intra-ductal fully covered self-expanding metal stent (ID-FCSEMS) to treat anastomotic biliary strictures (AS) after orthotopic liver transplantation (OLT). Surgical endoscopy 2017; 31: 1558-1563. DOI: 10.1007/s00464-016-5138-9. Epub 2016 Aug 29.
2. Al-Kawas FH. Biliary access during endoscopic retrograde cholangiopancreatography: How to precut and a word of caution! Journal of Gastroenterology and Hepatology (Australia) 2005; 20: 805-806. DOI: 10.1111/j.1400-1746.2005.03847.x
3. Aljahdli E. Management of distal malignant biliary obstruction. Saudi Journal of Gastroenterology 2018; 24: 71-72. DOI: 10.4103/sjg.SJG_611_17
4. Barresi L, Ligresti D, Traina M. A Challenging Diagnosis for a Biliary Stricture. Gastroenterology 2016; 150: 1537-1539. DOI: 10.1053/j.gastro.2016.02.024
5. Bhalla S, Law R. Endoscopic Management of Acute Cholecystitis Following Metal Stent Placement for Malignant Biliary Strictures: A View from the Inside Looking in. Clinical endoscopy 2019; 52: 209-211. DOI: 10.5946/ce.2019.097. Epub 2019 May 23.
6. Brijbassie A, Wang A, Hammerle C et al. Transpapillary vs. intraductal fully covered selfexpanding metal stent placement for malignant and benign biliary disease: Does it make a difference? Journal of Interventional Gastroenterology 2015; 5: 64-67. DOI: 10.7178/jig.192
7. Chang JH, Lee IS, Chun HJ et al. Usefulness of the rendezvous technique for biliary stricture after adult right-lobe living-donor liver transplantation with duct-to-duct anastomosis. Gut and liver 2010; 4: 68-75. DOI: 10.5009/gnl.2010.4.1.68. Epub 2010 Mar 25.
8. Chang JH, Lee IS, Chun HJ et al. Comparative study of rendezvous techniques in post-liver transplant biliary stricture. World Journal of Gastroenterology 2012; 18: 5957-5964. DOI: 10.3748/wjg.v18.i41.5957
9. Chantarojanasiri T, Kogure H, Hamada T et al. Usefulness of stent placement above the papilla, so-called, 'inside stent'. Gastrointestinal Intervention 2018; 7: 52-56. DOI: 10.18528/gii180013
10. ChiCtr. Comparative study for the efficacy of intraductal RFA + biliary stent placement or intraductal Iodine-125 seed + biliary stent placement by percutaneous transhepatic cholangial drainage for treating the patients with unresectable malignant biliary strictures. http://wwwwhoint/trialsearch/Trial2aspx?TrialID=ChiCTR1900020722 2019.
11. ChiCtr. Comparative study of the efficacy of intraductal RFA +stent placement by ERCP and percutaneous transhepatic approach in patients with unresectable malignant biliary strictures. http://wwwwhoint/trialsearch/Trial2aspx?TrialID=ChiCTR1900020693 2019.
12. Choi S, Jun C, Cho E et al. Feasibility of self expandable metal stent for preservation of sphincter of Oddi function in patients with common bile duct stones a pilot study. Hepatology International 2015; 9: S153-S154. DOI: 10.1007/s12072-015-9609-1
13. Choi W, Shim HJ. Newly modified plastic stent for transhepatic placement above the sphincter of Oddi in treatment of biliary anastomotic stricture after liver transplantation: Preliminary report of 7 patient. CardioVascular and Interventional Radiology 2019; 42: S251. DOI: 10.1007/s00270-019-02282-x
14. Choi W, Hur J. Abstract No. 579 Newly modified plastic stent for transhepatic placement above the sphincter of Oddi in treatment of biliary anastomotic stricture after liver transplantation: preliminary report of seven patients. Journal of Vascular and Interventional Radiology 2020; 31: S253. DOI: 10.1016/j.jvir.2019.12.640
15. Dawwas MF, Oppong KW, Webster GJ. Endoscopic assessment and management of biliary strictures. Frontline Gastroenterology 2016; 7: 170-175. DOI: 10.1136/flgastro-2015-100570
16. Douhara A, Ogawa H, Ozutsumi T et al. Inside plastic stent is useful and safety for malignant biliary stenosis including Bismuth IV. Journal of Gastroenterology and Hepatology (Australia) 2016; 31: 343. DOI: 10.1111/jgh.13540
17. García-Cano J, Álvarez-Burneo L, Taberna-Arana L. Difficulties encountered for removing fully covered self-expanding metal stents inserted in benign biliary conditions. United European Gastroenterology Journal 2013; 1: A494. DOI: 10.1177/2050640613502900
18. Gargouri D, Kochlef A, Ouekaa A et al. [Biliary stent occlusion]. La Tunisie medicale 2010; 88: 462-466.
19. Hasegawa H, Inatomi O, Morita Y et al. Appropriate therapeutic strategy for biliary stenting using threaded inside stents based on the location of the malignant obstruction. United European Gastroenterology Journal 2014; 2: A464. DOI: 10.1177/2050640614548980
20. Hatzidakis AA, Tsetis D, Chrysou E et al. Nitinol stents for palliative treatment of malignant obstructive jaundice: Should we stent the sphincter of Oddi in every case? CardioVascular and Interventional Radiology 2001; 24: 245-248. DOI: 10.1007/s00270-001-0030-x
21. Hayasaka N, Hayashi T, Ono M et al. Endoscopic placement of a large-bore covered self-expandable metallic stent for cholangitis caused by mucus from a pancreatic mucinous neoplasm. Endoscopy 2014; 46: E634-E635. DOI: 10.1055/s-0034-1377948
22. Hayashi K, Ikarashi S, Kohisa J et al. Endoscopic management of unresectable malignant hilar strictures using threaded inside stents versus metallic stents. Gastrointestinal Endoscopy 2018; 87: AB208.
23. Hyodo T, Yamanaka T. Endoscopic metallic stenting and intraductal ultrasonography in a case of bile duct cancer. Endoscopy 1998; 30: S97-S98. DOI: 10.1055/s-2007-1001414
24. Inatomi O, Bamba S, Shioya M et al. The efficacy and safety of threaded biliary plastic “inside-stent” for malignant hilar obstruction. Gastrointestinal Endoscopy 2011; 73: AB354. DOI: 10.1016/j.gie.2011.03.762
25. Inoue T, Kitano R, Ito K et al. Double radiofrequency ablation with metal stent placement for refractory benign biliary and pancreatic duct strictures. Journal of Gastrointestinal and Liver Diseases 2020; 29: 144. DOI: 10.15403/jgld-896
26. Inoue T, Suzuki Y, Okumura F et al. Intraductal placement of a fully covered metal stent with a long string for distal malignant biliary obstruction without endoscopic sphincterotomy: Prospective multi-center feasibility study. Digestive endoscopy :

official journal of the Japan Gastroenterological Endoscopy Society 2020; 32: 949-956. DOI: 10.1111/den.13614. Epub 2020 Mar 12.
27. Ishiwatari H, Hayashi T, Ono M et al. Newly designed plastic stent for endoscopic placement above the sphincter of Oddi in patients with malignant hilar biliary obstruction. Digestive endoscopy : official journal of the Japan Gastroenterological Endoscopy Society 2013; 25 Suppl 2: 94-99. DOI: 10.1111/den.12080.
28. Itoi T, Sofuni A, Itokawa F et al. Current status and issues regarding biliary stenting in unresectable biliary obstruction. Digestive endoscopy : official journal of the Japan Gastroenterological Endoscopy Society 2013; 25 Suppl 2: 63-70. DOI: 10.1111/den.12062.
29. Jo JH, Park BH. Suprapapillary versus transpapillary stent placement for malignant biliary obstruction: which is better? Journal of vascular and interventional radiology : JVIR 2015; 26: 573-582. DOI: 10.1016/j.jvir.2014.11.043. Epub 2015 Feb 10.
30. Jo J, Park B. Supra-papillary versus trans-papillary biliary stent for malignant biliary obstruction: Which is the better? Journal of Vascular and Interventional Radiology 2014; 25: S48. DOI: 10.1016/j.jvir.2013.12.115
31. Jprn U. Efficacy and Safety of inside stent for preoperative biliary drainage in patients with malignant hilar biliary obstruction: prospective,multicenter,single-arm study. http://wwwwhoint/trialsearch/Trial2aspx?TrialID=JPRN-UMIN000014111 2014.
32. Jprn U. Efficacy and safety of inside stent in patients with biliary stricture: a prospective multicenter study. http://wwwwhoint/trialsearch/Trial2aspx?TrialID=JPRN-UMIN000013291 2014.
33. Jprn U. The efficacy and safety of non-flared fully covered self expandable metalic stent for benign biliary duct stricture. http://wwwwhoint/trialsearch/Trial2aspx?TrialID=JPRN-UMIN000025027 2016.
34. Jprn U. Usefulness of Non-flared Intraductal Fully Covered Metallic Stent for Refractory Biliary Strictures after Living Donor Liver Transplantation. http://wwwwhoint/trialsearch/Trial2aspx?TrialID=JPRN-UMIN000021261 2016.
35. Jprn U. A Newly Modified Non-Flared Fully Covered Metallic Stent of 12 mm-Diameter with Long Lasso for Intraductal Placement in Patients with Malignant Biliary Stricture: feasibility Study. http://wwwwhoint/trialsearch/Trial2aspx?TrialID=JPRN-UMIN000013276 2014.
36. Jprn U. Plastic stent placement above the sphincter of Oddi ("inside-stent") for patients of malignant hilar biliary obstruction. A prospective phase II trial. http://wwwwhoint/trialsearch/Trial2aspx?TrialID=JPRN-UMIN000006779 2011.
37. Jprn U. Endoscopic treatment of malignant bile duct stricture; Comparative study of conventional stent vs inside stent. http://wwwwhoint/trialsearch/Trial2aspx?TrialID=JPRN-UMIN000006345 2011.
38. Jprn U. Inside-stent placement for malignant hilar obstruction. http://wwwwhoint/trialsearch/Trial2aspx?TrialID=JPRN-UMIN000004587 2011.
39. Jprn U. Randomized control trial of inside stenting using plastic stent and side by side stenting using 6mm full-covered self expandable metallic stent for endoscopic bilateral biliary drainage to malignant hilar biliary obstruction. http://wwwwhoint/trialsearch/Trial2aspx?TrialID=JPRN-UMIN000034036 2018.
40. Jprn U. Randomized controlled trial on efficacy of metal stents vs. inside plastic stents for unresectable malignant obstruction of the perihilar bile duct. http://wwwwhoint/trialsearch/Trial2aspx?TrialID=JPRN-UMIN000025760 2017.
41. Jprn U. Safety and efficacy of preoperative biliary drainage using plastic stent above the sphincter of Oddi (Inside-stent) in patients with malignant hilar biliary obstruction: a prospective study. http://wwwwhoint/trialsearch/Trial2aspx?TrialID=JPRN-UMIN000025463 2017.
42. Jprn U. The Usefulness of Newly Modified Non-flared Fully Covered Metal Stent of 12 mm in diameter Comparing with Conventional Stent for Periampullary Malignant Biliary Strictures. http://wwwwhoint/trialsearch/Trial2aspx?TrialID=JPRN-UMIN000030201 2017.
43. Jprn U. Efficacy of inside plastic stent placement for malignant hilar biliary obstruction -multicenter, randomized control trial. http://wwwwhoint/trialsearch/Trial2aspx?TrialID=JPRN-UMIN000036315 2019.
44. Jun CH, Park CH, Jeon J et al. Feasibility of self-expandable metal stents for preservation of sphincter of Oddi function in patients with common bile duct stones: A pilot study. Gastrointestinal Endoscopy 2015; 82: 719-723. DOI: 10.1016/j.gie.2015.01.034
45. Kaneko T, Sugimori K, Shimizu Y et al. Efficacy of plastic stent placement inside bile ducts for the treatment of unresectable malignant hilar obstruction (with videos). Journal of hepato-biliary-pancreatic sciences 2014; 21: 349-355. DOI: 10.1002/jhbp.41. Epub 2013 Oct 7.
46. Kawakami H, Kuwatani M, Eto K et al. Covered metallic stent for ischemic hilar biliary stricture. Digestive endoscopy : official journal of the Japan Gastroenterological Endoscopy Society 2012; 24 Suppl 1: 49-54. DOI: 10.1111/j.1443-1661.2012.01259.x.
47. Kogure H, Isayama H, Tsujino T et al. Plastic stent placement above the sphincter of Oddi ("inside stent") for biliary strictures. Gastroenterological Endoscopy 2011; 53: 1312-1319.
48. Kogure H, Tsujino T, Isayama H et al. Endoscopic management of biliary strictures after adult living donor liver transplantation with duct-to-duct reconstruction. Journal of Gastroenterology and Hepatology 2012; 27: 21-22. DOI: 10.1111/jgh.12005
49. Kogure H, Tsujino T, Isayama H et al. Endoscopic management of biliary strictures after adult living donor liver transplantation with duct-to-duct reconstruction. Gastrointestinal Endoscopy 2013; 77: AB317-AB318.
50. Koizumi M, Kumagi T, Kuroda T et al. Endoscopic stent placement above the sphincter of Oddi for biliary strictures after living donor liver transplantation. BMC gastroenterology 2020; 20: 92. DOI: 10.1186/s12876-020-01226-x.

51. Koizumi M, Kumagi T, Kuroda T et al. ENDOSCOPIC STENT PLACEMENT ABOVE THE SPHINCTER OF ODDI FOR BILIARY STRICTURES AFTER LIVING DONOR LIVER TRANSPLANTATION. Gastrointestinal Endoscopy 2019; 89: AB214. DOI: 10.1016/j.gie.2019.03.194
52. Kubota K, Hasegawa S, Ishii K et al. Inside stent and neoadjuvant chemotherapy can provide a chance of surgery in patients with symptomatic, initially unresectable klatskin tumors. Gastrointestinal Endoscopy 2015; 81: AB348. DOI: 10.1016/j.gie.2015.03.567
53. Kurita A, Kodama Y, Minami R et al. Endoscopic stent placement above the intact sphincter of Oddi for biliary strictures after living donor liver transplantation. Journal of gastroenterology 2013; 48: 1097-1104. DOI: 10.1007/s00535-012-0705-x. Epub 2013 Jan 17.
54. Lee HW, Moon JH, Lee YN et al. Usefulness of newly modified fully covered metallic stent of 12 mm in diameter and anti-migration feature for periampullary malignant biliary strictures: Comparison with conventional standard metal stent. Journal of Gastroenterology and Hepatology (Australia) 2019; 34: 1208-1213. DOI: 10.1111/jgh.14602
55. Lee J, Gwon DI, Ko GY et al. Biliary intraductal metastasis from advanced gastric cancer: radiologic and histologic characteristics, and clinical outcomes of percutaneous metallic stent placement. European radiology 2016; 26: 1649-1655. DOI: 10.1007/s00330-015-3995-6. Epub 2015 Sep 17.
56. Lee JK, Yang J, Park JK et al. Newly developed flower-type covered selfexpandable metal stent for preventing cholecystitis and pancreatitis: Physical properties. United European Gastroenterology Journal 2016; 4: A417-A418. DOI: 10.1177/2050640616663689
57. Lee DH, Yu JS, Hwang JC et al. Percutaneous placement of self-expandable metallic biliary stents in malignant extrahepatic strictures: indications of transpapillary and suprapapillary methods. Korean journal of radiology 2000; 1: 65-72. DOI: 10.3348/kjr.2000.1.2.65.
58. Leung JW, Lee W, Wilson R et al. Comparison of accessory performance using a novel ERCP mechanical simulator. Endoscopy 2008; 40: 983-988. DOI: 10.1055/s-2008-1077777. 2008 Dec 8.
59. Li Q, Qin Y, Yang F et al. Treatment experience of fully covered self-expandable metal stents in biliary stricture after liver transplantation. Liver Transplantation 2014; 20: S365. DOI: 10.1002/lt.23901
60. Liu Q, Khay G, Cotton PB. Feasibility of stent placement above the sphincter of Oddi ("inside-stent") for patients with malignant biliary obstruction. Endoscopy 1998; 30: 687-690. DOI: 10.1055/s-2007-1001389.
61. Moon JH, Choi HJ, Koo HC et al. Feasibility of placing a modified fully covered self-expandable metal stent above the papilla to minimize stent-induced bile duct injury in patients with refractory benign biliary strictures (with videos). Gastrointestinal endoscopy 2012; 75: 1080-1085. DOI: 10.1016/j.gie.2012.01.016. Epub 2012 Mar 7.
62. Moon JH, Choi HJ, Lee YN et al. A newly modified non-flared fully covered metallic stent of 12 mm-diameter with long lasso for intraductal placement in patients with malignant biliary stricture: Feasibility study. Gastrointestinal Endoscopy 2015; 81: AB399. DOI: 10.1016/j.gie.2015.03.705
63. Moon JH, Ko BM, Hong SJ et al. Biliary drainage with a modified fully covered self-expandable metallic stent for potentially resectable distal malignant biliary obstruction. Journal of Gastroenterology and Hepatology 2012; 27: 86. DOI: 10.1111/jgh.12006
64. Moon JH, Lee YN, Choi HJ et al. Usefulness of non-flared intraductal fully covered metallic stent for refractory biliary strictures after living donor liver transplantation: Interim results of a prospective multicenter trial. Gastrointestinal Endoscopy 2016; 83: AB134.
65. Morita S. [Biliary endoprosthesis of malignant biliary obstruction using expandable metallic stent--preliminary clinical evaluation]. Nihon Igaku Hoshasen Gakkai zasshi Nippon acta radiologica 1992; 52: 623-640.
66. Mukai T. Ways to improve stenting in unresectable malignant distal biliary obstruction: Stent design, intraductal placement, and protective role of an intact papilla? Digestive endoscopy : official journal of the Japan Gastroenterological Endoscopy Society 2020; 32: 891-893. DOI: 10.1111/den.13714. 2020 Jul 27.
67. Nct. Comparison Between Transpapillary and Suprapapillary Metal Stent. https://clinicaltrialsgov/show/NCT04503291 2020.
68. Nct. Intraintestinal Extended Biliary Stents Preventing Duodenobiliary Reflux in Patients With Biliary Stricture. https://clinicaltrialsgov/show/NCT04550819 2020.
69. Park SK, Moon JH, Choi HJ et al. Intraductal placement of modified fully covered metallic stent for distal malignant biliary obstruction. Journal of Gastroenterology and Hepatology (Australia) 2014; 29: 152. DOI: 10.1111/jgh.12766_2
70. Park JK, Moon JH, Lee YN et al. Intraductal placement of non-flared fully covered metallic stent for refractory anastomotic biliary strictures after living donor liver transplantation: Long-term results of prospective multicenter trial. Journal of Gastroenterology and Hepatology 2019; 34: 58. DOI: 10.1111/jgh.14862
71. Pécsi D, Vincze Á. Are Suprapapillary Biliary Stents Superior to Transpapillary Biliary Stents? Digestive Diseases and Sciences 2020; 65: 925-927. DOI: 10.1007/s10620-020-06068-x
72. Peixoto A, Moutinho-Ribeiro P, Macedo G. Degenerate intraductal papillary mucinous carcinoma with fish-mouth appearance of the papilla. Journal of Gastrointestinal and Liver Diseases 2017; 26: 113. DOI: 10.15403/jgld.2014.1121.262.pex
73. Sackmann M. Bile duct stenosis: Diagnosis and management by endoscopy. Journal of Gastrointestinal and Liver Diseases 2016; 25: 18.
74. Sandha GS, Roy A, Maniere T et al. Clinical utility of biliary metal stent use across a broad range of indications-interim results of a large prospective multi-centre Canadian registry. Gastrointestinal Endoscopy 2016; 83: AB250-AB251.

75. Sasahira N, Isayama H, Kogure H et al. Endoscopic management with inside stent for proximal benign biliary stricture after laparoscopic cholecystectomy. Digestive endoscopy : official journal of the Japan Gastroenterological Endoscopy Society 2012; 24 Suppl 1: 59-61. DOI: 10.1111/j.1443-1661.2012.01271.x.
76. Sato T, Kogure H, Isayama H et al. Endoscopic treatment of biliary strictures after adult living donor liver transplantation with duct-to-duct reconstruction. Digestive Endoscopy 2017; 29: 153. DOI: 10.1111/den.12775
77. Sato T, Kogure H, Nakai Y et al. Long-term outcomes of endoscopic treatment for duct-to-duct anastomotic strictures after living donor liver transplantation. Liver international : official journal of the International Association for the Study of the Liver 2019; 39: 1954-1963. DOI: 10.1111/liv.14219.
78. Shin J, Park JS, Jeong S et al. Comparison of the clinical outcomes of suprapapillary and transpapillary stent insertion in unresectable cholangiocarcinom a with biliary obstruction. United European Gastroenterology Journal 2019; 7: 496. DOI: 10.1177/205064061985467
79. Sung JY, Leung JWC, Shaffer EA et al. Ascending infection of the biliary tract after surgical sphincterotomy and biliary stenting. Journal of Gastroenterology and Hepatology 1992; 7: 240-245.
80. Takada R, Ikezawa K, Kiyota R et al. THE EFFICACY OF SELF-EXPANDABLE METALLIC STENT PLACEMENT ABOVE THE PAPILLA IN THE PATIENTS WITH DISTAL MALIGNANT BILIARY OBSTRUCTION. Gastrointestinal Endoscopy 2019; 89: AB212. DOI: 10.1016/j.gie.2019.03.189
81. Takahashi Y, Ito H, Inoue Y et al. Initial experience of endoscopic stent placement above the Oddi (inside-stent) for preoperative malignant biliary obstruction. Journal of Hepato-Biliary-Pancreatic Sciences 2017; 24: A297.
82. Taniguchi Y. Intraductal versus transpapillary fully covered self-expandable metal stent placement for malignant biliary strictures. Gastrointestinal Endoscopy 2018; 87: AB199.
83. Taniguchi Y. Intraductal vs. transpapillary fully covered metal stent placement for malignant biliary strictures. United European Gastroenterology Journal 2019; 7: 860. DOI: 10.1177/205064061985467
84. Walters DA, Geenen JE. Current role of endoscopic retrograde cholangiopancreatography in the management of benign pancreatic disease. Endoscopy 1998; 30: 174-181. DOI: 10.1055/s-2007-1001244
85. Yamaguchi A, Wada K, Moriuchi R et al. Intraductal plastic stent placement is an effective therapy for unresec table malignant biliary obstruction. United European Gastroenterology Journal 2019; 7: 572. DOI: 10.1177/205064061985467
86. Yamaguchi A, Miyasako Y, Takasago T et al. Intraductal plastic stent placement is an effective therapy for malignant biliary strictures. United European Gastroenterology Journal 2016; 4: A179. DOI: 10.1177/2050640616663689
87. Yamauchi H, Tadehara M, Kida M. Temporary non-flared fully covered self-expandable metal stent placement for refractory benign choledochojejunal anastomotic stricture. Digestive Endoscopy 2018; 30: 541-542. DOI: 10.1111/den.13069
88. Yang MJ, Lee GH, Kim JH et al. Hepatobiliary and Pancreatic: Management of a benign bilio-enteric stricture by a novel covered stent with a long lasso. Journal of Gastroenterology and Hepatology (Australia) 2019; 34: 1477. DOI: 10.1111/jgh.14747
89. Yoo JJ, Lee JK, Moon JH et al. Intraductal placement of non-flared fully covered metallic stent for refractory anastomotic biliary strictures after living donor liver transplantation: Long-term results of prospective multicenter trial. Journal of gastroenterology and hepatology 2020; 35: 492-498. DOI: 10.1111/jgh.14831. 2019 Sep 10.
90. Zeissig S, Schmelz R, Brückner S et al. Direct endoscopy and diagnosis of adenocarcinoma following metal stent-based drainage of a pancreatic cyst. Endoscopy 2018; 50: E72-E73. DOI: 10.1055/s-0043-124182
91. Brijbassie A. Transpapillary vs. intraductal fully covered selfexpanding metal stent placement for malignant and benign biliary disease: does it make a difference? Journal of Interventional Gastroenterology 2015; 5. DOI: 10.7178/jig.192
92. Cho JN HJ, Kim HG, Shin IH, Park SH, Moon JH, Kim JH, Lee DH, Maetani I, Maguchi H, Hanada K, Yasuda I, Itoi T, Isayama H, Lee D. Prospective randomized trial comparing covered metal stent placed above and across the sphincter of oddi in malignant biliary obstruction. Gastrointestinal endoscopy 2013; 77.
93. Cosgrove N, Siddiqui AA, Adler DG et al. A Comparison of Bilateral Side-by-Side Metal Stents Deployed Above and Across the Sphincter of Oddi in the Management of Malignant Hilar Biliary Obstruction. J Clin Gastroenterol 2017; 51: 528-533. DOI: 10.1097/mcg.0000000000000584
94. Inatomi O, Bamba S, Shioya M et al. Threaded biliary inside stents are a safe and effective therapeutic option in cases of malignant hilar obstruction. BMC Gastroenterology 2013; 13: 31. DOI: 10.1186/1471-230X-13-31
95. Kobayashi N, Watanabe S, Hosono K et al. Endoscopic inside stent placement is suitable as a bridging treatment for preoperative biliary tract cancer. BMC Gastroenterology 2015; 15: 8. DOI: 10.1186/s12876-015-0233-2
96. Kubota K, Hasegawa S, Iwasaki A et al. Stent placement above the sphincter of Oddi permits implementation of neoadjuvant chemotherapy in patients with initially unresectable Klatskin tumor. Endosc Int Open 2016; 4: E427-433. DOI: 10.1055/s-0042-102246
97. Lee HM, JH; Choi, HJ; Lee, YN; Lee, TH; Choi, MH; Cha, SW; Cho, YD; Park, SH. MODIFIED FULLY COVERED SELF-EXPANDABLE METAL STENT VERSUS PLASTIC STENT FOR PREOPERATIVE BILIARY DRAINAGE IN PATIENTS WITH RESECTABLE MALIGNANT BILIARY OBSTRUCTION. Endoscopy 2018; 44 - 45.
98. Pedersen FM, Lassen AT, Schaffalitzky de Muckadell OB. Randomized trial of stent placed above and across the sphincter of Oddi in malignant bile duct obstruction. Gastrointestinal endoscopy 1998; 48: 574-579. DOI: 10.1016/s0016-5107(98)70038-0.
99. Shin J, Park JS, Jeong S et al. Comparison of the Clinical Outcomes of Suprapapillary and Transpapillary Stent Insertion in Unresectable Cholangiocarcinoma with Biliary Obstruction. Digestive diseases and sciences 2020; 65: 1231-1238. DOI: 10.1007/s10620-019-05859-1 10.1007/s10620-019-05859-1. Epub 2019 Oct 4.

100. Takada R, Ikezawa K, Kiyota R et al. Self-expandable metallic stent placement above the papilla without endoscopic sphincterotomy in patients with distal malignant biliary obstruction. Endoscopy international open 2020; 8: E753-e760. DOI: 10.1055/a-1135-8437. 2020 May 25.
101. Taniguchi Y. Intraductal vs. transpapillary fully covered metal stent placement for malignant biliary strictures. GASTROINTESTINAL ENDOSCOPY 2020; 91.
102. Uchida N, Tsutsui K, Ezaki T et al. Estimation of the stent placement above the intact sphincter of Oddi against malignant bile duct obstruction. Journal of Gastroenterology 2005; 40: 291-296. DOI: 10.1007/s00535-004-1535-2
103. Yamaguchi A, Moriuchi, R., Wada, K., Tao, K., Konishi, H., Miura, R., ... & Kouno, H. Intraductal Plastic Stent Placement is an Effective Therapy for Unresectable Malignant Hilar Biliary Obstruction. JOURNAL OF GASTROENTEROLOGY AND HEPATOLOGY 2019; 34: 175-175.
